# A New Method for the Visualization of Living Dopaminergic Neurons and Prospects for Using It to Develop Targeted Drug Delivery to These Cells

**DOI:** 10.3390/ijms23073678

**Published:** 2022-03-27

**Authors:** Victor Blokhin, Alina V. Lavrova, Sergey A. Surkov, Eduard R. Mingazov, Natalia M. Gretskaya, Vladimir V. Bezuglov, Michael V. Ugrumov

**Affiliations:** 1 Koltzov Institute of Developmental Biology of the Russian Academy of Sciences, 26 Vavilov Street, 119334 Moscow, Russia; victor.blokhin@hotmail.com (V.B.); surkov@eimb.ru (S.A.S.); ed.mingazov89@gmail.com (E.R.M.); 2 Shemyakin–Ovchinnikov Institute of Bioorganic Chemistry, Russian Academy of Sciences, Miklukho-Maklaya 16/10, 117997 Moscow, Russia; alinalavrova1@gmail.com (A.V.L.); natalia.gretskaya@gmail.com (N.M.G.); vvbez2013@yandex.ru (V.V.B.)

**Keywords:** Parkinson’s disease, mice, humans, cell culture, mesencephalon, dopaminergic neurons, LUHMES cells, dopamine transporter, GBR-12909, targeted therapy

## Abstract

This is the first study aiming to develop a method for the long-term visualization of living nigrostriatal dopaminergic neurons using 1-(2-(bis(4-fluorophenyl)methoxy)ethyl)-4-(3-phenylpropyl)piperazine-BODIPY (GBR-BP), the original fluorescent substance, which is a derivative of GBR-12909, a dopamine uptake inhibitor. This method is based on the authors’ hypothesis about the possibility of specifically internalizing into dopaminergic neurons substances with a high affinity for the dopamine transporter (DAT). Using a culture of mouse embryonic mesencephalic and LUHMES cells (human embryonic mesencephalic cells), as well as slices of the substantia nigra of adult mice, we have obtained evidence that GBR-BP is internalized specifically into dopaminergic neurons in association with DAT via a clathrin-dependent mechanism. Moreover, GBR-BP has been proven to be nontoxic. As we have shown in a primary culture of mouse metencephalon, GBR-BP is also specifically internalized into some noradrenergic and serotonergic neurons, but is not delivered to nonmonoaminergic neurons. Our data hold great promise for visualization of dopaminergic neurons in a mixed cell population to study their functioning, and can also be considered a new approach for the development of targeted drug delivery to dopaminergic neurons in pathology, including Parkinson’s disease.

## 1. Introduction

Dopamine is one of the most widely distributed classical neurotransmitters in the brain, involved in the regulation of important functions such as reproduction, motor behavior, and mental health [1,2]. The regulation of motor function is by dopaminergic neurons of the nigrostriatal system and the regulation of reproduction by dopaminergic neurons of the tuberoinfundibular system [1,3,4,5,6]. Degeneration of the dopaminergic neurons of the tuberoinfundibular system and the nigrostriatal system leads to the development of neurodegenerative diseases, hyperprolactinemia, and Parkinson’s disease (PD). Hyperprolactinemia responds well to treatment, while PD is a severe, incurable disease [7,8,9,10,11].

For many years (up to 30), PD develops at the preclinical stage, without the manifestation of characteristic motor symptoms, which are used for the diagnosis of PD [12,13]. Motor symptoms first appear when 50–60% of dopaminergic neurons in the substantia nigra die and dopamine levels in the striatum, a key link in the regulation of motor function, decrease by 70–80% [7,14,15]. In addition to dopaminergic neurons, noradrenergic and serotonergic neurons of the brain are involved in the regulation of motor behavior, and in PD they begin to degenerate earlier than dopaminergic neurons [16,17].

The current symptomatic pharmacotherapy for PD is based on the systemic administration of dopamine agonists and precursors, primarily L-3,4-dihydroxyphenylalanine. However, the effectiveness of this treatment is very limited, mainly due to its late initiation and the fact that it does not target neurodegeneration, the fundamental cause of the disease [11,18]. Moreover, over time, symptomatic therapy becomes toxic, as manifested in the appearance of drug-induced dyskinesia [19,20,21].

It follows from the above that the development of early (preclinical) diagnosis and preventive neuroprotective treatment for PD is among the highest priorities in neurology. This could slow down the degeneration of dopaminergic neurons and prolong the preclinical (asymptomatic) stage of the disease—the period of normal physical and social activity for patients [11]. The success of such developments is possible due to obtaining new fundamental knowledge about the molecular mechanisms of the pathogenesis of PD, with an emphasis on the degeneration of nigrostriatal dopaminergic neurons.

There are many morphological and molecular biological methods for studying dopaminergic neurons on sections of fixed nervous tissue or in a homogenate [22]. However, there are still no methods for the long-term study of the functioning of living dopaminergic neurons in a mixed cell population, for example, in the substantia nigra, which contains, in addition to dopaminergic neurons, other neurons and glial cells [23]. Therefore, the goal of this study has been to develop a new method for the long-term specific visualization of living nigral dopaminergic neurons. As a dye, we have used fluorescent substances that we have recently synthesized on the basis of GBR-12909, a dopamine uptake inhibitor [24]. The following objectives were set: (i) to synthesize fluorescent substances for staining living dopaminergic neurons, using GBR-12909 as a template, (ii) to determine whether synthesized fluorescent substances stain embryonic mesencephalic dopaminergic neurons in primary culture and freshly prepared slices of the substantia nigra of adult animals, (iii) to determine whether and to what extent the staining of dopaminergic neurons is specific, stable in time, nontoxic, and realized through a dopamine transporter (DAT)-dependent mechanism, and (iv) to determine if our fluorescent substances stain other monoaminergic neurons (serotoninergic and noradrenergic), as well as nonmonoaminergic neurons. The last (fifth) objective of this study was to consider the obtained data on staining of living dopaminergic neurons as a prerequisite for the development of targeted drug delivery to dopaminergic neurons in PD. Indeed, based on long-term experience studying dopaminergic neurons in the brain in health and disease, including PD [11,25,26], we considered the possibility of targeted drug delivery to dopaminergic neurons in the brain via a DAT-dependent mechanism (Personal communication, M. Ugrumov, 13 April 2016). The prospect of successful development of targeted drug delivery to monoaminergic neurons, especially dopaminergic neurons, is more realistic than to any other cell. This is due to the presence only on the surface of monoaminergic neurons of a specific protein—the monoamine transporter, which is internalized through clathrin-dependent endocytosis [27,28,29].

## 2. Results

### 2.1. Synthesis of GBR-BODIPY and GBR-ATTO

**Figure 1:**
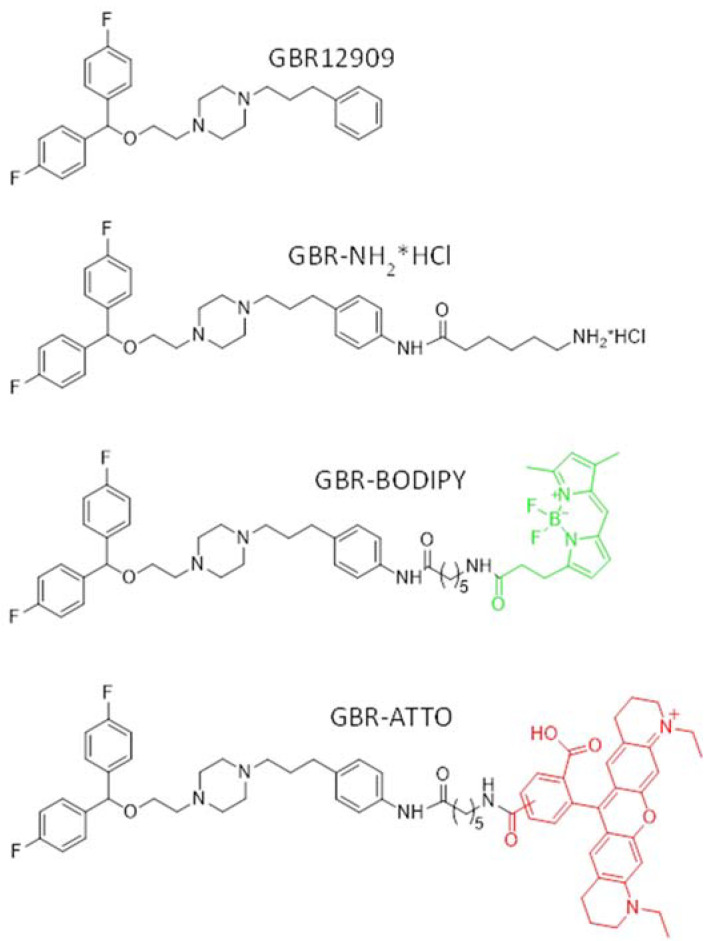
Structures of 1-(2-(bis(4-fluorophenyl)methoxy)ethyl)-4-(3-phenylpropyl)piperazine (GBR-12909) and its modified (GBR-NH_2_*HCl) and fluorescent analogs GBR–BODIPY (GBR-BP) (green—BODIPY fluorophore) (Modified from [30]) and GBR-ATTO (red—ATTO fluorophore).

Fluorescent analogs of GBR-12909 were synthesized using commercially available BODIPY-C3 acid and ATTO-565-NHS (succinimide ester) by addition to compound GBR-NH2*HCl, as previously described [24] (Figure 1 and Figure S1).

The yield of the target compounds was about 40%, with a purity of at least 95% according to high-performance liquid chromatography (Figures S2 and S3).

The fluorescence spectra of GBR-BP and BODIPY-C3 are practically indistinguishable (Absorption = λmax 505 nm, Fluorescence Emission λmax = 512 nm; Absorption* = λmax 505 ± 3 nm, Fluorescence Emission* λmax = 511 ± 4 nm, respectively) (Figure S4a). For GBR-ATTO, there was a hypsochromic shift in the fluorescence spectrum compared to the original label (Absorption λmax = 564 nm, Fluorescence Emission λmax = 590 nm; Absorption (according to the manufacturer’s data) λmax = 556 nm, Fluorescence Emission* λmax = 577 nm, respectively) (Figure S4b). A similar effect was observed when modifying proteins with this fluorescent label (Atto Tec. Available online: https://www.atto-tec.com/fileadmin/user_upload/Katalog_Flyer_Support/Procedures.pdf (accessed on 1 January 2022)). It should be noted that, for both fluorescent analogs of GBR-12909, no intramolecular interaction of fluorescent labels with the benzene and GBR-12909 rings was observed, leading to fluorescence quenching or changes in ultraviolet (UV) spectra, which was also confirmed by modeling the structures of these analogs (Figure S5). 

### 2.2. Immunocytochemical Detection of Protein Markers of Monoaminergic Neurons in the Primary Cell Culture of the Mouse Embryonic Brain and in Differentiated LUHMES Cells

**Figure 2:**
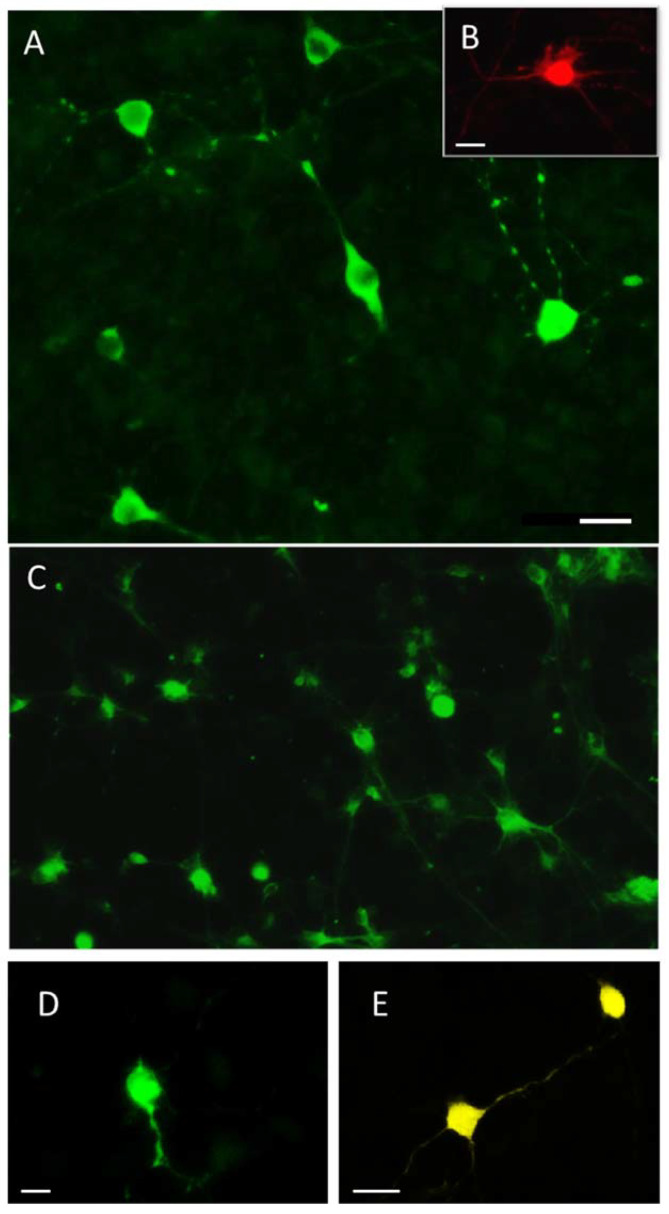
Primary mouse mesencephalon culture containing numerous tyrosine hydroxylase-immunopositive neurons (**A**) and rare 5-hydroxytryptamine-immunopositive neurons (**B**), as well as a primary mouse metencephalon culture containing numerous dopamine-β-hydroxylase-immunopositive neurons (**C**), as well as rare dopamine transporter-immunopositive neurons (**D**) and 5-hydroxytryptamine-immunopositive neurons (**E**). Objective magnification = ×20 (Plan-Neufluar). Bars = 25 µm for (**A**,**C**,**E**); 15 µm for (**B**); 10 µm for (**D**).

One week after culturing mouse embryonic mesencephalon, we found numerous tyrosine hydroxylase (TH)-immunopositive neurons and rare 5-hydroxytryptamine (5-HT)-immunopositive neurons (Figure 2A,B), whereas dopamine β-hydroxylase (DBH)-immunopositive neurons were not found.

After culturing mouse embryonic metencephalon for one week, numerous dopamine-β-hydroxylase(DBH)-immunopositive neurons and rare 5-HT-immunopositive neurons were found (Figure 2C). Although DAT immunopositive neurons were also detected in this culture (Figure 2D,E), their numbers were extremely low (two cells in all studied materials). All the above mentioned immunostained cells were represented by bipolar or multipolar neurons. 

**Figure 3:**
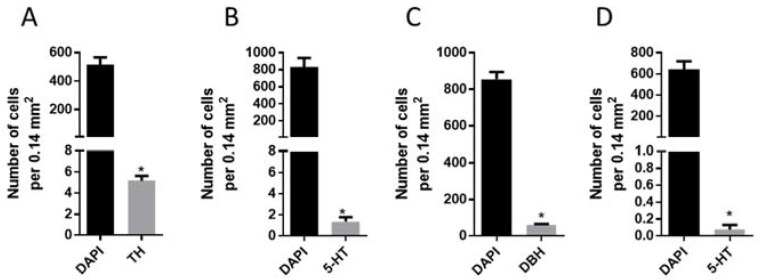
Mean number of all cells labeled with DAPI-stained nuclei in a primary culture of mesencephalon (**A**,**B**) and metencephalon (**C**,**D**) in mice in randomly selected culture areas of 0.14 mm², as well as the average number of mesencephalic neurons immunopositive for tyrosine hydroxylase (TH) (**A**) or 5-hydroxytryptamine (5-HT) (**B**), as well as of metencephalic neurons immunopositive for dopamine-β-hydroxylase (DBH) (**C**) or 5-HT (**D**). * *p* ˂ 0.05.

Mesencephalic and metencephalic cell cultures were used to count the total number of cells identified by DAPI-stained nuclei, per 0.14 mm^²^ of culture, as well as the number of mesencephalic cells immunopositive for tyrosine hydroxylase and 5-HT, and metencephalic cells immunopositive for DBH and 5-HT (Figure 3).

This quantitative analysis also made it possible to estimate the ratio between different populations of immunopositive neurons (in %) in primary cultures of mesencephalon and metencephalon. So, if mesencephalic TH-immunopositive neurons and 5-HT-immunopositive neurons together are taken to be 100%, then the population of TH-immunopositive neurons is 85.7% and that of 5-HT-immunopositive is 14.3%. The same calculation of the proportion of different populations of immunopositive neurons in metencephalon showed that the fraction of DBH-immunopositive neurons is 99.88% and that of 5-HT-immunopositive neurons is 0.12%.

**Figure 4:**
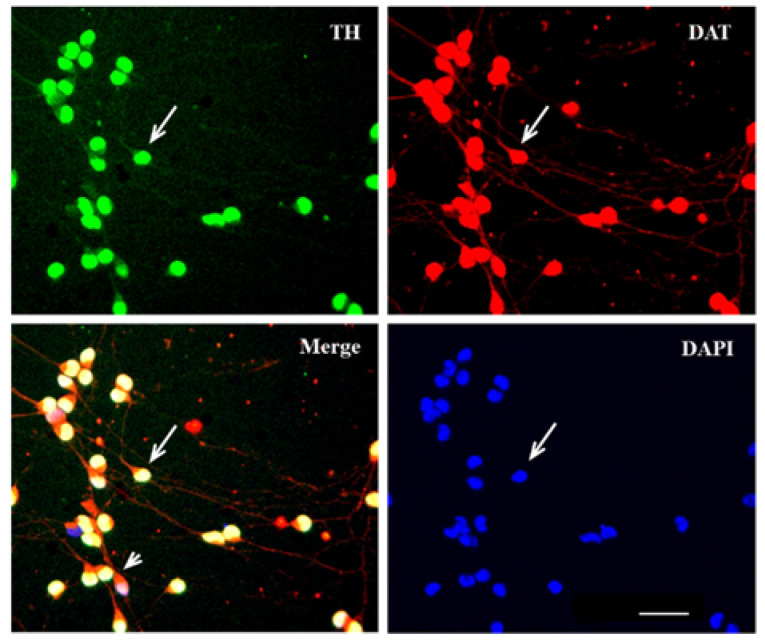
LUHMES cells double-immunostained for tyrosine hydroxylase (TH, green. AlexaFluor 488) and dopamine transporter (DAT, red, AlexaFluor546), with nuclei stained with DAPI (blue). Merge-colocalization of TH, DAT and DAPI (yellow) Arrows indicate cells immunostained for TH and DAT and stained with DAPI; the arrowhead indicates the transition zone of the cell body to the cell process, occupied by DAT. Objective magnification = ×20 (Plan-Neufluar). Bar = 50 µm.

Mono-immunostaining of LUHMES cells (human embryonic mesencephalic cells) a week after the onset of their differentiation using antibodies to TH, DAT, DBH, and 5-HT, and with additional staining of cell nuclei with DAPI, showed that almost all of these cells are bipolar round or oval TH- and DAT-immunopositive neurons (Figure 4). The intensity of immunostaining of cell processes did not differ from that of cell bodies. According to double-immunolabeling, practically all LUHMES cells are both TH- and DAT-immunopositive (Figure 4). The cell bodies are characterized by a slightly different distribution of TH-immunopositive and DAT-immunopositive materials. TH-immunopositive material occupies most of the cell body, with the exception of the transition area between the cell body and one of the processes. This area contains mainly DAT-immunopositive material. The prevalence of DAT-immunopositive material is also a characteristic of cell processes (Figure 4).

### 2.3. Living Mouse Embryonic Mesencephalic Cells Stained with GBR-BP and GBR-ATTO, and Living LUHMES Cells Stained with GBR-BP

**Figure 5:**
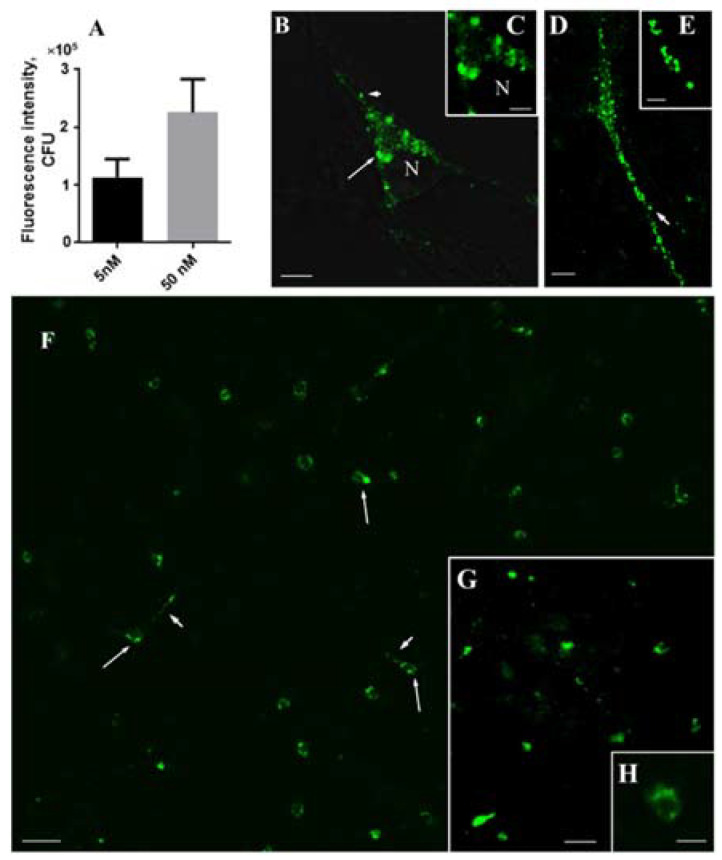
Conventional and confocal fluorescence microscopy of GBR-BP-stained living cells of the primary culture of the mouse embryonic mesencephalon (7th day of cultivation) and living cells of the substantia nigra (vibratome sections) of an adult mouse. (**A**) Fluorescence intensity of cells of the embryonic mesencephalon when incubated with GBR-BP at a concentration of 5 nM or 50 nM. In all experiments (**B**–**H**), GBR-BP was used at a concentration of 50 nM. (**B**,**C**) GBR-BP deposition in the neuron cell bodies in rounded vacuoles of various sizes (mesencephalic culture); (**D**,**E**) GBR-BP deposition in the neuron processes in small rounded vesicular structures (mesencephalic culture); (**F**) general view of a primary mesencephalon culture; (**G**,**H**) GBR-BP-stained cells in the vibratome sections of adult mouse substantia nigra. Objective magnification = ×64 (**B**–**E**); objective magnification = ×10 (**F**). arrow, cell body; arrowhead, cell processes. Bars = 5 µm for (**B**,**E**); 2.5 µm for (**C**); 50 µm for (**F**); 20 µm for (**G**); 10 µm for (**H**). CFU, cell fluorescence unit; N, nucleus.

Living mouse embryonic mesencephalic cells were examined on the 7th day of cultivation after incubation for 1 h with GBR-BP at a concentration of 5 nM or 50 nM at 37 °C. The staining intensity of cells after incubation with GBR-BP at a concentration of 5 nM was much lower than after incubation with GBR-BP at a concentration of 50 nM (Figure 5A). Therefore, in further experiments, we used GBR-BP at a concentration of 50 nM.

**Figure 6:**
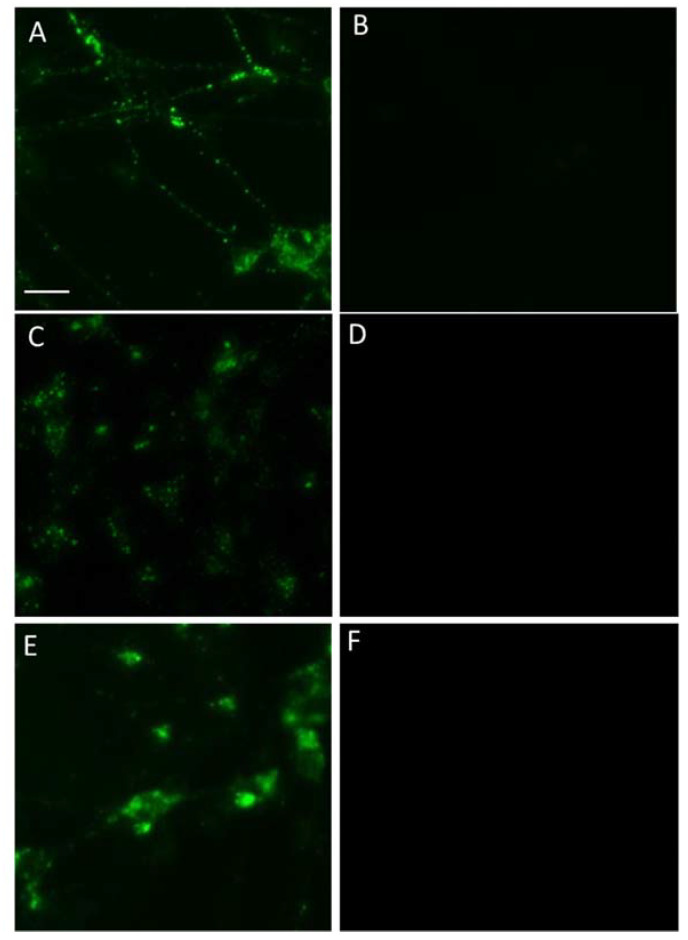
Mouse mesencephalon (**A**,**B**) and metencephalon (**C**,**D**) primary cultures and LUHMES cells (**E**,**F**) after incubation with GBR-BP (50 nM, 60 min) (**A**,**C**,**E**) or after pre-incubation with GBR-12909 (10 µM, 15 min) and subsequent co-incubation (60 min) with GBR-BP (50 nM) and GBR-12909 (10 µM) (**B**,**D**,**F**). Objective magnification = ×20. Selective fluorescent staining with GBR-BP is prevented by GBR-12909, a monoamine transporter inhibitor. Bar = 25 µm for all photos.

According to our data, numerous mouse embryonic mesencephalic cells were stained after 1 h of incubation with GBR-BP at a concentration of 50 nM (Figure 5B–F). They looked like bipolar or multipolar neurons, containing rounded fluorescent inclusions 0.5 to 2 µm in diameter (Figure 5B–E). Although the fluorescent inclusions in the cell bodies varied in number and size, large inclusions located around the nucleus predominated (Figure 5B,C). Unlike the cell bodies, the cell processes contained small granular inclusions no more than 0.8 μm in size (Figure 5D,E). In the control, after pre-incubation of cells with GBR-12909 and subsequent incubation with GBR-BP and GBR-12909 at 37 °C, no fluorescent cells were found (Figure 6A,B).

Incubation of mouse mesencephalic cells with GBR-ATTO for 60 min at 37 °C resulted in uniform staining of individual cells and their conglomerates, which have little resemblance to neurons. After pre-incubation of mesencephalic cells with GBR-12909 and subsequent incubation with GBR-ATTO and GBR-12909 at 37 °C, the cells remained stained. Based on these results, only GBR-BP was used in further studies. Importantly, no fluorescent cells were detected after incubation of mouse mesencephalic cells with GBR-BP at 4 °C. Mouse mesencephalic cells also did not stain after co-incubation with GBR-BP and sucrose at 37 °C.

Time-lapse photography of the mouse mesencephalic cells during incubation with GBR-BP for 17 min showed that the cells began to stain immediately after the onset of incubation, and the intensity of staining increased gradually over time (Video S1). First, small fluorescent inclusions ≥0.3 µm appeared, mainly in the cell processes. Then the fluorescent inclusions moved along the processes towards the cell bodies. During transportation, the fluorescent inclusions increased in size, reaching a maximum of about 2 µm in the cell body. The data obtained from time-lapse photography are consistent with the data obtained from one-time photography of mesencephalic cells after incubation with GBR-BP for 60 min. In the control, the administration of GBR-12909 before and during incubation with GBR-BP prevents the staining of mouse mesencephalic cells, which is seen in both types of registration (Figure 6A,B). In addition, no fluorescent mesencephalic cells were observed with time-lapse photography during incubation with GBR-BP and sucrose at 37 °C for 17 min. Staining of mesencephalic cells with GBR-BP was also prevented by incubation with GBR-BP at 4 °C.

In addition to staining cells of the primary culture of the mouse embryonic mesencephalon with GBR-BP, we also stained with GBR-BP cells in vibratome sections of the substantia nigra of adult mice (Figure 5G,H).

**Figure 7:**
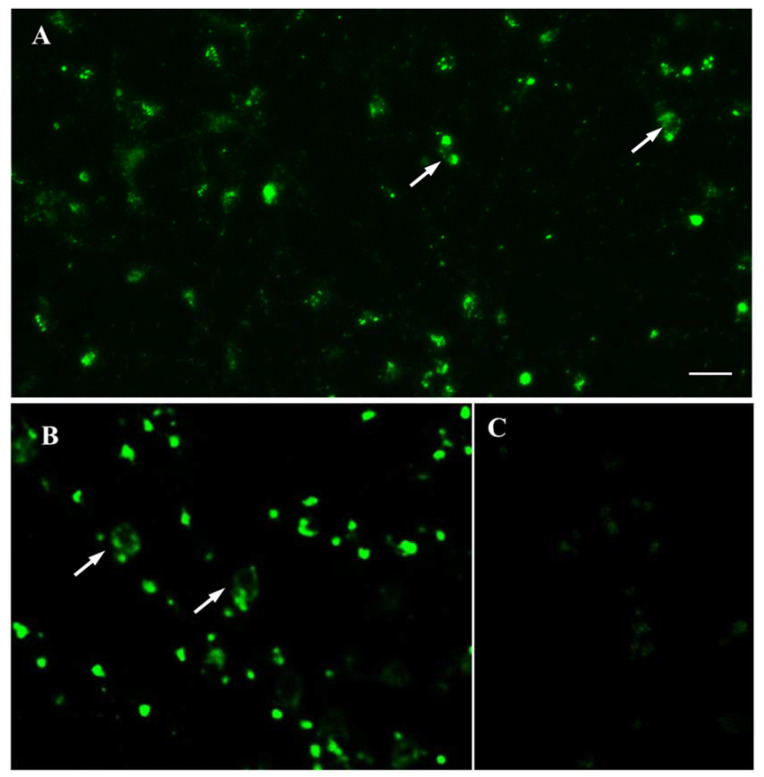
Fluorescence microscopy of living mouse metencephalic cells (7th day of culturing) (**A**) and LUHMES cells (7th day of differentiation) (**B**) following 60 min incubation (staining) with GBR-BP at a concentration of 50 nM, as well as of LUHMES cells 72 h after the incubation with 50 nM GBR-BP for 60 min (**C**). Objective magnification = ×20 (Plan-Neufluar). Arrow, GBR-BP-stained cell. Bar = 25 µm for all photos.

As in the primary mouse embryonic mesencephalon culture, numerous fluorescent cells were observed in the primary mouse embryonic metencephalon culture after 1 h of incubation with 50 nM GBR-BP at 37 °C (Figure 7A).

However, fluorescent metencephalic cells were not visible after pre-incubation with GBR-12909 and subsequent co-incubation with GBR-12909 and GBR-BP (Figure 6C,D).

After 1 h of incubation of differentiated LUHMES cells with 50 nM GBR-BP at 37 °C, almost all cells were stained (Figure 7B). However, fluorescent cells were absent after preincubation with GBR-12909 and then incubation with GBR-12909 and GBR-BP (Figure 6E,F). The staining of GBR-BP LUHMES cells lasted for 72 h. Indeed, at the end of this period the fluorescence intensity was barely noticeable (Figure 7C).

### 2.4. Identification of Protein Markers of Monoaminergic Neurons in Fixed Cells of Mouse Embryonic Mesencephalon and Metencephalon after Preliminary Staining of These Living Cells with GBR-BP

**Figure 8:**
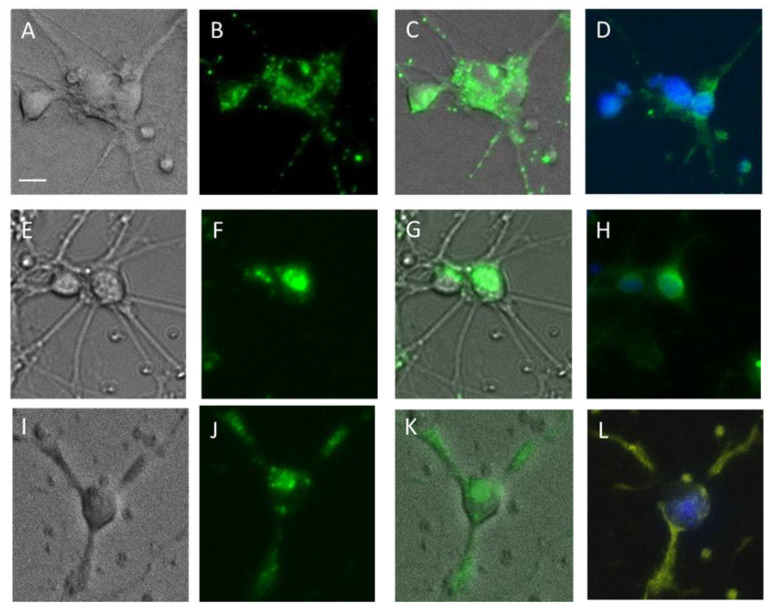
Microphotos of primary cell culture of the mouse mesencephalon (**A**–**D**) and metencephalon (**E**–**L**) (7th day of cultivation) after the following procedures: (i) phase contrast photography (**A**,**E**,**I**); (ii) incubation with 50 nM GBR-BP and photography of GBR-BP-stained fluorescent cells (**B**,**F**,**J**); (iii) merging the images obtained in “i” and “ii” (**C**,**G**,**K**); (iv) fixation with 4% paraformaldehyde with loss of GBR-BP fluorescence followed by fluorescent immunostaining of mesencephalic cells for tyrosine hydroxylase ((TH); dilution 1:500) (Abcam, ab19353) (**D**) and metencephalic cells for dopamine-β-hydroxylase ((DBH); dilution 1:500) (Chemicon, ab1542)) (**H**) or 5-hydroxytryptamine (5-HT; dilution 1:750) (Sigma, S5575) (**L**) and DAPi staining of neuronal nuclei. Objective × 20 (Plan-Neufluar). Bar = 50 µm.

According to the obtained data, most mesencephalic cells prestained with GBR-BP, after fixation, were immunopositive for TH (Figure 8D), whereas metencephalic cells were immunopositive for DBH and 5-HT (Figure 8H,L).

### 2.5. Assessment of the Possible Toxic Effect of GBR-BP on Mouse Mesencephalic Cells and Human LUHMES Cells

**Figure 9:**
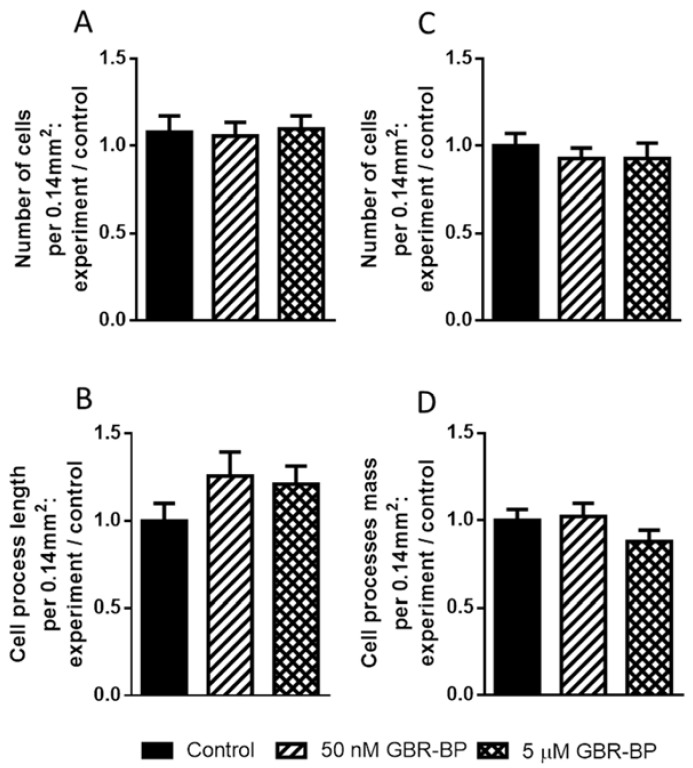
The ratio of the number of tyrosine hydroxylase-immunopositive cells in the primary culture of the mouse mesencephalon (**A**) and the total length of their processes (**B**), as well as the ratio of the number of LUHMES cells stained with calcein (**C**) and the mass of their processes (**D**) after 12-h incubation in a medium containing 50 nM or 5 µM GBR-BP (experiment) to these parameters when these cells were incubated without GBR-BP (control). In all cases, there are no significant differences (*p* > 0.05). The power is: 0.3642 for (**A**), 0.272 for (**B**), 0.1869 for (**C**), and 0.3431 for (**D**).

After a 12-h incubation of a primary culture of mouse embryonic mesencephalon with GBR-BP at a concentration of 50 nM or 5 μM, the total number of TH-immunopositive peroxidase-labeled neurons and the length of their processes per neuron were the same as in the control (Figure 9).

**Figure 10:**
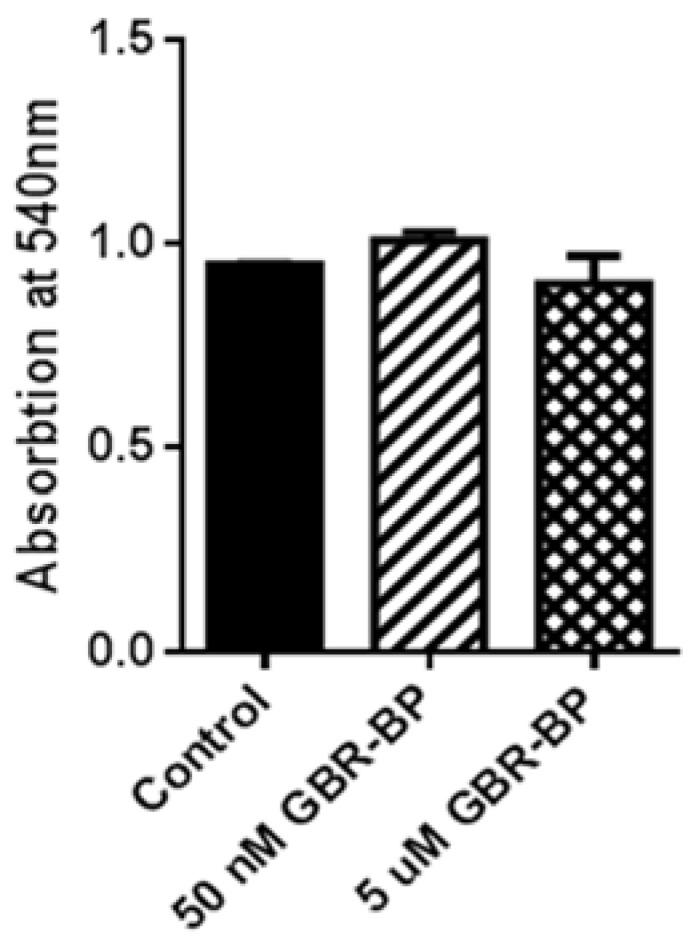
Optical density of formazan, a derivative of 3-(4,5-dimethyl-2-thiazolyl)2,5-diphenyl-2H-tetrazolium bromide, in LUHMES cells after 24-h incubation with GBR-BP at a concentration of 50 nM or 5 μM and in the control (without GBR-BP). No significant differences were observed (*p* > 0.05).

Given that all LUHMES cells express TH (see Section 2.2), TH immunostaining was meaningless. Therefore, we used nonimmunostained, but calcein-stained LUHMES cells to calculate their number and the total length of processes per cell. As in the case of mesencephalic TH-immunopositive cells, we did not find any difference in the number of LUHMES cells and the total length of their processes per cell after 12-h incubation with GBR-BP, compared to the control (Figure 9). In addition, using a 3-(4,5-dimethyl-2-thiazolyl)2,5-diphenyl-2H-tetrazolium bromide (MTT) assay, we found no changes in the optical density of LUHMES cell extracts after 24 h of incubation with GBR-BP (Figure 10).

## 3. Discussion

According to the paradigm of translational medicine, the improvement or development of methods for the diagnosis and treatment of diseases, including PD, should be based on fundamental knowledge of the cellular and molecular mechanisms of pathogenesis. Proceeding from this idea, the goal of this study was to develop a new method for the long-term visualization of living dopaminergic neurons, which would allow us to study the functioning of these neurons in a mixed population with other neurons and non-neuronal cells.

At the first stage of developing a method for imaging dopaminergic neurons (the first task), we synthesized two fluorescent DAT ligands using GBR-12909, the most specific inhibitor of monoamine uptake, including dopamine, as a template [30,31,32]. We assumed that fluorescent labeling of the DAT ligand molecule would make it possible to observe its penetration into neurons, intraneuronal movement, and deposition. For this, we synthesized an analog of GBR-12909 containing an aminohexanoic acid residue attached by an amide bond to the amino derivative GBR-12909: 6-amino-N-(4-(3-(4-(2-(bis(4-fluorophenyl) methoxy)ethyl)piperazin-1-yl)propyl)phenylamine. The specified amino derivative GBR-12909 was described earlier [33]. The transformation of the aromatic amino group was difficult due to its weak reactivity. The compound GBR-NH_2_*HCl we synthesized contains an aliphatic amino group separated from the main inhibitor molecule by a hydrocarbon chain of five methylene units, which makes it possible to sterically separate the ligand part from the cargo [24]. In this study, we used two fluorescent analogs of GBR-12909 containing different fluorophores, BODIPY and ATTO-565, to test how the structure of the additional molecular fragment would affect the ligand properties of GBR-12909.

Based on the above, we turned to the second objective of this study: to test whether GBR-BP and GBR-ATTO molecules, in combination with different fluorescent dyes, will stain dopaminergic neurons, and to determine the mechanisms of this staining. This was done using cells of a primary culture of the mouse embryonic mesencephalon, the anlage of the substantia nigra. This and previous studies have shown that the mesencephalon culture contains mostly neurons expressing the dopaminergic phenotype, including DAT [34,35]. According to this study, both synthesized substances, GBR-BP and GBR-ATTO, stain the living cells of the mouse embryonic mesencephalon culture. However, only GBR-BP staining could be prevented by GBR-12909, showing that GBR-BP is internalized into dopaminergic neurons via a DAT-dependent mechanism. In contrast, the staining of the cells with GBR-ATTO was not prevented by preincubation with GBR-12909 and subsequent incubation with GBR-ATTO and GBR-12909. From a comparison of the results, it follows that, unlike GBR-BP, GBR-ATTO stains mesencephalic cells nonspecifically, apparently due to its greater hydrophobicity. Indeed, the calculated hydrophobicity index (CLog P) for GBR-BODIPY (7.4711) was significantly lower than for GBR-ATTO (12.2281). Therefore, in our further studies, we used only GBR-BP for staining living dopaminergic neurons. Using slices of the substantia nigra of an adult mouse, we showed that GBR-BP is internalized not only into differentiating embryonic dopaminergic neurons, but also into differentiated dopaminergic neurons of adult animals.

As in the case of GBR-12909 [30,31], we noted the dose dependence of the specific delivery of GBR-BP into dopaminergic neurons using mouse embryonic mesencephalic cells. Indeed, the fluorescence intensity of cells in the primary culture of the mouse mesencephalon and, hence, the level of GBR-BP delivery into dopaminergic neurons was significantly lower when using GBR-BP at a concentration of 5 nM than at a concentration of 50 nM. Surprisingly, the staining of mesencephalic cells with GBR-BP at a concentration of 5 nM was rather low, although, according to Andersen [31], GBR-12909 is already selectively captured by dopaminergic synaptosomes at a concentration of 1 nM. Proceeding from the above data, GBR-BP was used in further experiments at a concentration of 50 nM.

The third objective of this study was to obtain additional evidence for the specific internalization of GBR-BP into dopaminergic neurons in the mouse embryonic mesencephalon culture. In this context, we have shown that 78% of mesencephalic cells stained with GBR-BP are immunopositive for TH, a marker of catecholaminergic neurons [36]. Taking into account that, according to the literature data [34,35] and our immunocytochemical data, the embryonic mesencephalon culture contains mainly dopaminergic neurons and only rare serotoninergic neurons, we consider TH-immunopositive cells in this brain region as dopaminergic neurons.

We believe that cells stained with GBR-BP but immunonegative for tyrosine hydroxylase are serotonergic neurons found in primary midbrain culture, as well as some dopaminergic neurons containing tyrosine hydroxylase at differentiation below the resolution of immunocytochemistry. The latter idea is supported by observations of neurons expressing the TH gene but being TH-immunonegative [26,37].

Our evidence of the internalization of GBR-BP into the dopaminergic neurons of the mesencephalon culture via a DAT-dependent mechanism is indirectly confirmed by the temperature dependence of this process, which is also characteristic of the uptake of dopamine and 4-(4-(dimethylamino) styryl)-N-methylpyridinium iodide (ASP+), a high-affinity fluorescent ligand of the membrane transporters of monoamines—dopamine, noradrenaline, and serotonin [38,39,40]. In fact, GBR-BP hardly stains mesencephalic cells at 4 °C. For understanding the mechanism of GBR-BP internalization into dopaminergic neurons, the fact of prevention of mesencephalic dopaminergic neuron staining by GBR-BP after preincubation with sucrose is of particular interest. Considering that sucrose is one of the inhibitors of clathrin-dependent endocytosis [41,42], our data suggest that GBR-BP is internalized into dopaminergic neurons in association with DAT by endocytosis due to a clathrin-dependent mechanism. This is consistent with the opinion that DAT internalization is also a clathrin-dependent process [29,41]. It follows from the above that the mechanism of GBR-BP delivery into dopaminergic neurons is fundamentally different from that for ASP+, which penetrates to dopaminergic neurons via DAT and accumulates in mitochondria [40].

To elucidate the mechanisms of internalization and the fate of GBR-BP in dopaminergic neurons, it was necessary to determine its localization in neurons. In this study, it has already been shown through confocal microscopy that GBR-BP is mainly stored in the cell bodies of dopaminergic neurons in large oval vacuole-like structures. However, the resolution of the confocal microscopy is too low to identify subcellular inclusions in which GBR-BP is accumulated. This issue is expected to be clarified in the next electron microscopic study. Thus, our data obtained in following the second and third objectives suggest that the DAT-dependent internalization mechanism of GBR-BP can be used, albeit with caution, for targeted drug delivery to dopaminergic neurons. Considering that the above evidence for the internalization of GBR-BP into dopaminergic neurons via a DAT-dependent mechanism was obtained using a culture of mouse embryonic dopaminergic neurons, it was necessary to confirm that GBR-BP can also be selectively delivered to human dopaminergic neurons. To achieve this (fourth) objective, we used LUHMES immortalized cells of the human embryonic mesencephalon, which, after stopping proliferation and subsequent differentiation, express a dopaminergic phenotype, including the ability to specifically capture dopamine with DAT [43]. It is important to emphasize that, in our study, all LUHMES cells, after differentiation, express a dopaminergic phenotype, being immunopositive for both TH and DAT. Moreover, we have shown that GBR-BP stains practically all living differentiated LUHMES cells. As in the case of mouse embryonic mesencephalic neurons, the staining of LUHMES cells was prevented by GBR-12909. Finally, our results show that GBR-BP is internalized via a DAT-dependent mechanism, not only into the dopaminergic neurons of the primary culture of mouse embryonic mesencephalon, but also into the dopaminergic neurons of LUHMES cells.

After obtaining evidence that GBR-BP is specifically delivered via a DAT-dependent mechanism into the dopaminergic neurons of the mouse and human mesencephalon (LUHMES cells), it was necessary to elucidate whether GBR-BP would also be captured into the noradrenergic and serotoninergic neurons, as well as into the nonmonoaminergic neurons. The internalization of GBR-BP into noradrenergic and serotonergic neurons was a priori highly probable. Indeed, in previous studies, it was shown that dopamine is already specifically captured into dopaminergic neurons at a rather low concentration (10^−10^ M–10^−11^ M), whereas at a higher concentration (˃10^−7^ M), it is also captured into noradrenergic and serotoninergic neurons [44,45]. Therefore, the fourth objective of this study was to determine whether GBR-BP, at a concentration of 50 nM, would be captured not only into dopaminergic neurons, but also into noradrenergic and serotonergic neurons. It was particularly important to test whether GBR-BP can be nonspecifically captured into nonmonoaminergic neurons. In order to solve this problem, we used a primary cell culture of mouse embryonic metencephalon, the brain stem anlage containing, according to our immunocytochemical data, noradrenergic (DBH-immunopositive) neurons, serotonergic (5-HT-immunopositive) neurons, and nonmonoaminergic (TH-, DBH- and 5-HT-immunonegative) neurons. We have shown that, as in the case of the mouse embryonic mesencephalon primary culture, many living cells in the metencephalon culture are stained with GBR-BP. Internalization of GBR-BP into metencephalic cells via a monoamine transporter-dependent mechanism, as in the case of mesencephalic cells, was confirmed by preventing the staining of these cells using GBR-12909. Subsequent immunocytochemical analysis of fixed metencephalic cells showed that the number of cells prestained with GBR-BP is half the number of DBH-immunopositive cells. These data probably mean that, at the concentration we used, GBR-BP is internalized to a much greater extent into dopaminergic neurons than into noradrenergic neurons. In the primary culture of the metencephalon, we did not observe cells stained with GBR-BP, but lacking proteins—markers of monoaminergic neurons. From the above data, it follows that GBR-BP at a concentration of 50 nM is mainly internalized into dopaminergic neurons through a DAT-dependent mechanism, although it can also be captured to a lesser extent by other monoaminergic neurons.

Before we synthesized GBR-BP on the template of GBR-12909, only one high-affinity fluorescent ligand of monoamine membrane transporters was known: ASP+ [46,47,48]. A fundamentally important difference between ASP+ and GBR-BP is that ASP+ cannot be used as a potential drug carrier, since it is similar in structure to 1-methyl-4-phenylpyridinium (MPP+), a specific dopaminergic neuron toxin. Indeed, MPP+ is specifically captured by monoamine transporters into monoaminergic neurons, mainly into dopaminergic neurons, causing oxidative stress and neuron death [47]. Evidence of the toxic effect of ASP+ includes its accumulation in the mitochondria of monoaminergic neurons [47], as well as a drop in the membrane potential of neurons 3–4 h after incubation with ASP+ [48].

We have obtained evidence that, in contrast to ASP+, GBR-BP does not exert a toxic effect on dopaminergic neurons. This conclusion derives from the fact that the number of dopaminergic neurons and the length of their neurites do not change after a 12-h incubation of mouse mesencephalon primary culture and LUHMES cells with GBR-BP compared to the control (0.05% DMSO). This conclusion was strongly supported by the fact that we did not observe any changes in LUHMES cells with a MTT test even after 24 h of incubation with GBR-BP. Under the same incubation but with MPP+ (concentration 5 μM or 10 μM, 12-h incubation) instead of GBR-BP, a massive degeneration was observed in LUHMES cells [49,50].

The evidence obtained in this study that GBR-BP is internalized into dopaminergic neurons and is nontoxic opens up great prospects for the use of long-term (up to 72 h) imaging of living dopaminergic neurons to study their functional activity in a mixed population of cells in animals with normal health and pathologies, including PD. At the same time, this approach seems to be promising for the further development of targeted drug delivery to dopaminergic neurons. It should be noted that DAT has long attracted the attention of neuropharmacologists [30,31,32], since its inhibition allows to increase the concentration of dopamine in the synaptic cleft and thereby prolong its action. A natural DAT inhibitor is cocaine, a substance isolated from *Erythroxylum coca* leaves [51]. Despite the fact that numerous analogs of cocaine with improved pharmacological characteristics have been synthesized, the mental side effects prevent their use for the treatment of patients. From this point of view, substances from the benztropine family turned out to be much more promising DAT inhibitors, especially those that have a piperazine core instead of a tropane fragment. Thus, GBR-12909 showed the highest affinity (IC50 = 4.3 nM) and selectivity for DAT [30]. The physiological efficacy of this compound has been demonstrated in experiments on monkeys when simulating PD using 1-methyl-4-phenyl-1,2,3,6-tetrahydropyridine, a proneurotoxin of dopaminergic neurons [52]. Moreover, GBR-12909 (vanoxeamine) was considered in previous studies as a potential drug that can have a neuroprotective effect on dopaminergic neurons through D2 autoreceptors in some diseases, including cerebral ischemia, and reduce drug (cocaine) dependence [53,54].

Although the development of targeted drug delivery to specific organs and cells [55] is one of the most pressing problems in pharmacology and pharmacotherapy, progress in this field is limited [55]. When developing targeted drug delivery to the brain, difficulties arise due to the presence of the blood–brain barrier [56]. The list of technologies that are currently used or being developed for targeted drug delivery to the brain includes: (i) intranasal administration of drugs and their delivery along the concentration gradient via the cranial nerves and vascular bundles into the brain, bypassing the blood–brain barrier [57,58], (ii) local reversible opening of the blood–brain barrier using ultrasound [59], (iii) drug administration into the cerebral ventricles near the target brain regions [60], and (iv) delivery of drugs directly to the pathological regions in the brain [61,62].

Among the above methods of targeted drug delivery to the brain, the one that is most widely used in neurology and neuro-oncology is the fourth, which ensures the delivery of anticarcinogenic drugs to brain tumors [63]. This approach is based on the binding of molecular constructs, mainly cytotoxins, to specific protein molecules on the surface of tumor cells, usually receptors, and their internalization. However, the therapeutic potential of this approach is limited due to the absence of specific proteins on the surface of most cancer cells. In carcinogenesis, this is exacerbated by permanent mutations in genes encoding surface proteins [56,63]. In this context, our study opens up unique prospects for the development of targeted drug therapy of neurodegenerative diseases, including PD. We proceed from two prerequisites that would allow us to develop this technology. First, among all brain cells, only monoaminergic, dopaminergic, noradrenergic, and serotonergic neurons have a specific surface protein, an outer loop of the plasma membrane monoamine transporter [41,64]. Second, monoamine transporters, like some receptors, are internalized into monoaminergic neurons by endocytosis, which is a crucial mechanism for the transfer of substances from the extracellular environment into cells [41,65,66]. We believe that the targeted delivery of neuroprotectors or their translational factors to nigrostriatal dopaminergic neurons can be of particular importance for the preventive treatment of PD at the early (preclinical) stage. Such therapy will, if not stop, then at least slow down the death of dopaminergic neurons and, thereby, prolong the preclinical (asymptomatic) stage of the disease, and, hence, the period of normal physical and social activity for patients. Targeted delivery of neuroprotectors into dopaminergic neurons can also significantly increase the effectiveness of current symptomatic therapy based on the systemic use of dopamine agonists or dopamine metabolic precursors [11,67,68].

## 4. Materials and Methods

### 4.1. GBR-BP and GBR-ATTO Synthesis 

For the synthesis of GBR-BP and GBR-ATTO, we used the following materials: acetonitrile, ethyl acetate, triethylamine, sodium chloride, sodium sulfate (Chimmed, Moscow, Russia), chloroform (Ekos-1, Moscow, Russia), methanol, isobutylchloroformate (Fluka, Buchs, Swithzerland), BODIPY™ FL C5 (Lumiprobe, Hannover, Germany), and ATTO565-NHS (ATTO-TEC, Siegen, Germany).

Thin-layer chromatography (TLC) was performed on Kieselgel 60 F254 plates (Merck, Darmstadt, Germany) in chloroform–methanol (10:1) and chloroform–methanol–water (65:25:4) systems. Column chromatography was performed on Silica Gel 60 (Merck, Germany). The purity of the synthesized substances was determined on a “Milichrom A-02” liquid microcolumn chromatograph (EcoNova, Moscow, Russia), high-performance liquid chromatography (HPLC) column ProntoSil 120-5-C18 AQ 5 μm (2.0 × 75 mm) (Bischoff, Leonberg, Germany). For the target and intermediate compounds obtained, the purity was at least 90%. UV spectra were recorded on an Agilent Technologies Cary 60 UV-Vis UV spectrometer (Agilent Technologies, Santa Clara, CA, USA); fluorescence spectra were recorded on a Hitachi F-4000 spectrofluorometer (Hitachi, Tokyo, Japan); mass spectra were obtained on an Orbitrap Q-Exactive Plus Thermo Scientific mass spectrometer (USA). The degree of hydrophobicity was estimated by calculating the octanol–water distribution coefficient of ClogP using the ChemDraw Professional 16.0 software (Perkin Elmer, Waltham, MA, USA). 3D images of GBR12909 fluorescent analog molecules were created using the PyMOL Molecular Graphics System, Version 2.5.2 (Schrödinger, Cambridge, MA, USA).

Fluorescent analogue of GBR-12909–GBR-BP (3) (Figure S1). To a solution of 0.9 mg (3.1 μmol) of BODIPY-C3 acid in 0.2 mL of acetonitrile, 63 μL (5.1 μmol) of triethylamine and 60 μL (5.1 μmol) of isobutyl chloroformate were added and the mixture was incubated at 4 °C for 10 min. The reaction mixture was diluted with ethyl acetate (20 mL) and water (5 mL) and sequentially washed with water (2 × 10 mL) and saturated sodium chloride solution (10 mL). The organic phase was dried with anhydrous Na_2_SO_4_. The drying agent was filtered off and the filtrate was evaporated. Yield: 1.1 mg (91%, red crystals) of compound (5), used further without additional purification.

To a solution of 2.3 mg (3.4 μmol) of compound (2) [24] in 0.2 mL of acetonitrile, we added 6.6 μL (4.7 μmol) of triethylamine and 1.1 mg (2.8 μmol) of the above prepared compound (5) and stirred it at room temperature for 1 h. The solvent was evaporated and the residue was dissolved in 0.5 mL of chloroform and chromatographed on a silica gel column in a chloroform–methanol system in gradient mode. The fractions containing the product were combined and evaporated, and the residue was dried under vacuum. Yield: 0.93 mg (32%, green crystals). TLC: Rf 0.5 (chloroform–methanol, 10:1) mass spectrum, m/z: 853.476 [M+H]+, 833.478 [M-F]+ (Figure S3a).

Preparation of GBR-ATTO (4), the fluorescent analog of GBR-12909) (Figure S1). To 1 mg (1.5 μmol) compound (2) in 61 μL of acetonitrile we added 3 μL (2.2 μmol) of triethylamine. The resulting solution was transferred to a solution of 0.83 mg (1.2 μmol) of succinimidyl ester ATTO-565 in 139 μL of acetonitrile and the mixture was incubated at room temperature for 12 h. The solvent was evaporated and the residue was dissolved in 0.3 mL of chloroform and chromatographed on a silica gel column in a chloroform–methanol system in gradient mode. The fractions containing the product were combined and evaporated, and the residue was dried under a vacuum. Yield: 0.5 mg (40%, violet crystals). TLC: Rf 0.9 chloroform–methanol–water, 65:25:4) mass spectrum, m/z: 1071.56 [M] +, 1072.56 [M+H] +, 1073.57 [M+2H]+ (Figure S3b).

### 4.2. Animals 

In this study, we used C57Bl/6 mice, males and females: aged 2–2.5 months and weighing 22–26 g, purchased from the “Pushchino” SPF animal facility (Pushchino, Moscow, Russia). The animals were kept at 21–23 °C and on a 12-h day–night cycle with free access to food and water.

**Figure 11:**
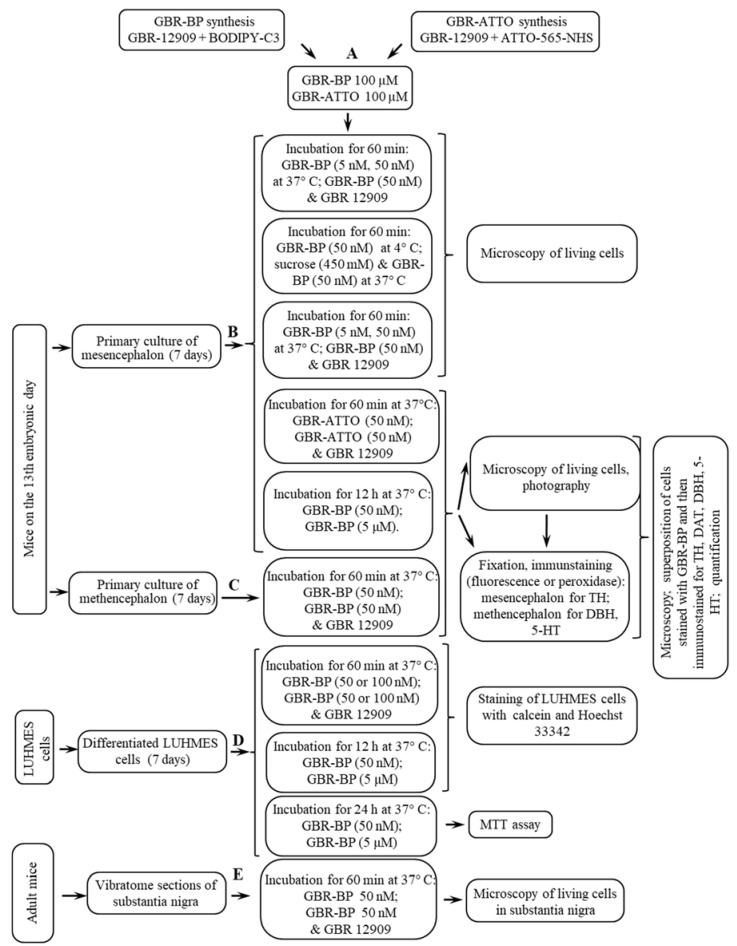
Design of experiments on cells of a primary culture of the mouse embryonic mesencephalon and metencephalon, the immortalized cells of the human mesencephalon (LUHMES) after differentiation, as well as on vibratome sections of the substantia nigra of adult mice: (**A**) GBR-BP and GBR-ATTO synthesis; (**B**) staining of mesencephalic living cells with 1-(2-(bis(4-fluorophenyl)methoxy)ethyl)-4-(3-phenylpropyl)piperazine–BODIPY (GBR-BP) and GBR-ATTO; (**C**) staining of metencephalic living cells with GBR-BP; (**D**) staining of living LUHMES cells with GBR-BP; (**E**) staining of nigral living cells with GBR-BP. Fixation of GBR-BP-prestained cells and immunostaining for proteins—markers of dopaminergic, noradrenergic, and serotonergic neurons: tyrosine hydroxylase (TH), dopamine-β hydroxylase (DBH), and 5-hyroxytryptamine (5-HT) (serotonin), respectively.

**Figure 12:**
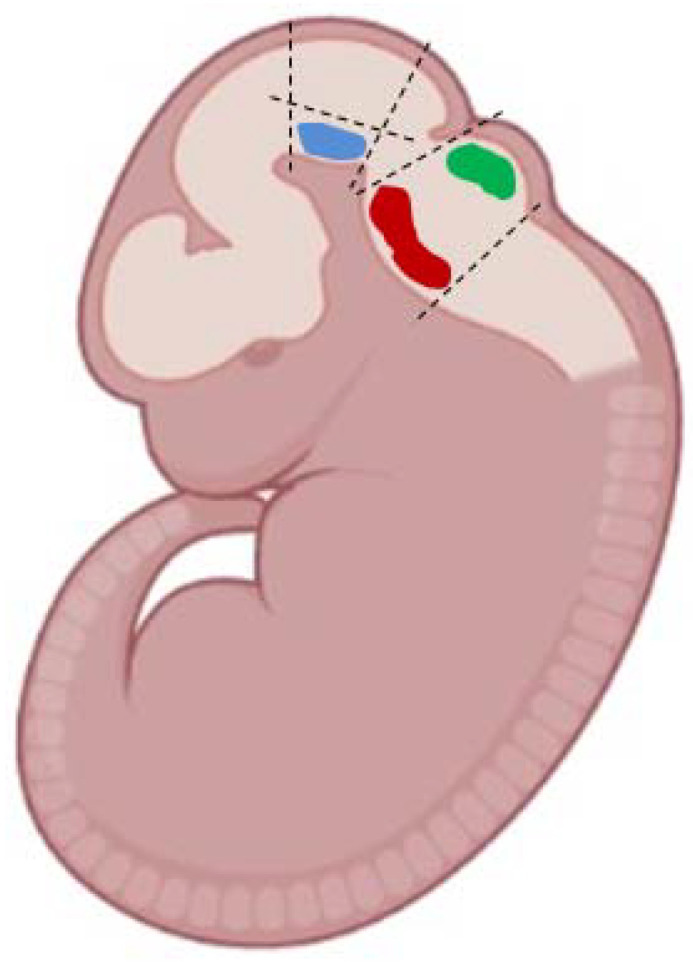
Scheme for obtaining the ventral mesencephalon (blue spot)—the anlage of the substantia nigra, the metencephalon—the anlage of the raphe nucleus (red spot), and the locus coeruleus (green spot) in mice on the 13th embryonic day. Modified from [70,71].

Some males were decapitated under isoflurane anesthesia (SomnoSuite, Kent Scientific, Torrington, CT, USA), and their brains removed for the immediate preparation of vibratome sections (Figure 11). Other animals were used to produce a dated pregnancy. For this, males were placed in cages with females in the evening, and the next morning, if a vaginal plug was found, this day was considered the first day of pregnancy. On day 13 of pregnancy, females underwent dislocation of the cervical vertebrae, then laparotomy was performed and the uterus with the embryos was excised. It was washed with cooled Ca^2+^ and Mg^2+^ free Hanks’ buffer (Gibco, Waltham, MA, USA) with the addition of gentamicin and amphotericin (Gibco, Waltham, MA, USA) [69]. Then the embryos were removed from the uterus, decapitated, and their brains dissected. In the above medium and under the control of a binocular loupe (Zeiss, Oberkochen, Germany), we cut out the ventral mesencephalon from the brain of some embryos, and the metencephalon from others (Figure 12), as described previously [70,71].

All procedures with animals were carried out in accordance with the requirements of the NIH Guide for the Care and Use of Laboratory Animals and the Bioethics Committee of the Koltzov Institute of Developmental Biology of the Russian Academy of Sciences (protocol No. 3 from 10 September 2020 and protocol No. 44 from 24 December 2020).

### 4.3. Preparation of Vibratome Sections of the Substantia Nigra of Adult Mice 

Under the control of a binocular loupe (Zeiss, Oberkochen, Germany), the substantia nigra was cut out of the brain of adult animals at the coordinates–5.2 to 0 mm from Bregma, in accordance with the mouse brain atlas [72]. The obtained block of nervous tissue was mounted on a table placed in a chamber with cooled (4 °C) Krebs-Ringer buffer (KRB) of the following composition (mM): NaCl 120, KCl 4.8, CaCl_2_ 2.0, MgSO_4_ 1.2, NaHCO_3_ 25, D-glucose 10.1, HEPES 20, and ascorbic acid 0.13 (all from Sigma-Aldrich, St. Louis, MO, USA), pH 7.4. Then, serial sections of the substantia nigra, 70 µm thick, were prepared using a vibratome (Microm HM 650 V, Thermo Scientific, Waltham, MA, USA).

### 4.4. Preparation of Embryonic Mouse Mesencephalon and Embryonic Mouse Metencephalon Primary Cell Culture 

The ventral mesencephalon and metencephalon excised from the brain on the embryonic day 13 (Figure 12) were dissociated according to a previously published protocol [73]. For this purpose, the excised brain pieces were placed into a microtube containing Dulbecco’s Modified Eagle Medium and Nutrient Mixture F-12 (DMEM/F12) with serum-free supplements B-27 (2% *v/v*) and 0.5 mM L-glutamine (Gibco, Waltham, MA, USA) and dissociated by pipetting. The resulting cell suspension was filtered through a nylon filter (70-μm pores) (Corning, New York, NY, USA) and centrifuged at 200× *g* for 5 min at +4 °C. The supernatant was removed and the residue was resuspended in neurobasal medium with serum-free supplements B-27 (2% *v/v*), 0.5 mM L-glutamine, 100 ng/mL nerve growth factor, and 10 ng/mL mouse fibroblast growth factor with the addition of gentamicin (50 μg/mL) (all reagents from Gibco, Waltham, MA, USA). The cell suspension was stained with 0.4% trypan blue (Sigma-Aldrich, St. Louis, MO, USA) and the stained (dead) cells were counted in the hemocytometer. The survival rate was 80–90%.

The cells in the culture medium were then seeded into 24-well plates pretreated with poly-L-lysine (Corning, New York, NY, USA), with 400,000 cells per well, and cultured in an atmosphere with 5% CO_2_ at 37 °C. The culture medium was changed every two days. On the fourth day of cultivation, we added 10 μM cytarabine (Sigma-Aldrich, St. Louis, MO, USA) to the medium for 24 h to suppress proliferation of glial cells. On the 7th day of cultivation, the cells were used for research.

### 4.5. LUHMES Cells Differentiation 

In addition to the primary culture of mouse embryonic mesencephalic and metencephalic cells, we used cells of the commercial LUHMES cell line (CRL-2927, ATCC, Manassas, VA, USA). These cells, obtained from the mesencephalon of eight-week-old human embryos, were immortalized with the retroviral vector “LINX” containing the v-myc oncogene under the control of a tetracycline promoter, which allows the cells to proliferate. LUHMES cells, placed into a DMEM/F12 medium with 10% fetal bovine serum and 10% DMSO (Gibco, Waltham, MA, USA, Sigma-Aldrich, St. Louis, MO, USA), were frozen in liquid nitrogen and stored at −70 °C.

Before starting the experiments, LUHMES cells were thawed at 37 °C in a DMEM/F12 growth medium (1:1) (Gibco, Waltham, MA, USA) additionally containing 1% serum-free supplement N-2 (Gibco, Waltham, MA, USA), 2 mM glutamine, 15 mM HEPES (Gibco, Waltham, MA, USA), and 20 ng/mL growth factor b-FGF (Gibco, Waltham, MA, USA). The volume ratio of the thawed cell suspension and the growth medium was 1:10. The cell suspension was centrifuged at 200× *g* for 10 min, and, after removing the supernatant, we resuspended the residue (the cells) in 1 mL of growth medium, counting the number of cells in the hemocytometer. The resulting proliferating cells were seeded into Petri dishes with a diameter of 60 mm, precoated with polyornithine and then with fibronectin (Sigma-Aldrich, St. Louis, MO, USA), or into standard 24-well plates with 15-mm wells with 10 μM GBR-12909 and 50 nM GBR-BP at 37 °C (Figure 11D).

Proliferating cells were further cultured in a growth medium with supplements (see above) at 37 °C in an atmosphere with 5% CO_2_ until the cells covered at least 80% of the inner surface of the bottom of the Petri dish. After that, the medium was removed and the cells were washed three times with Dulbecco’s phosphate-buffered saline (PBS) solution (Gibco, Waltham, MA, USA) without Ca^2+^ and Mg^2+^. The cells were then separated from the plastic by 5-min incubation in a DMEM/F12 medium with 1% N-2, 2 mM glutamine, 15 mM HEPES, and 20 ng/mL growth factor b-FGF, as well as with 1 μg/mL tetracycline (Sigma-Aldrich, St. Louis, MO, USA), 0.05% trypsin, and 4.8 mM EDTA (Gibco, Waltham, MA, USA). Thereafter, the cell suspension was centrifuged for 10 min at 1000 rpm. We then resuspended the residue (the cells) by pipetting in a DMEM/F12 medium with N-2, 2 mM glutamine, 15 mM HEPES, 20 ng/mL growth factor b-FGF, and 1 µg/mL tetracycline (Sigma-Aldrich, St. Louis, MO, USA). The number of cells in the resulting suspension was counted by a hemocytometer.

The obtained LUHMES cells were seeded into a plastic dish coated with polyornithine and fibronectin (Sigma-Aldrich, St. Louis, MO, USA) at a density of 5 × 10^4^ cells per cm^2^. The cells were cultured in a predifferentiation medium of the same composition as the growth medium, containing 1 μg/mL tetracycline, but without b-FGF, for 4 to 12 h, depending on the rate of cell attachment to a fibronectin substrate, at 37 °C in an atmosphere with 5% CO_2_. The attachment of cells to the substrate was monitored under a microscope. Nonadherent cells were removed by washing with PBS. The cells attached to the substrate were incubated for seven days in the differentiation medium DMEM/F12 (Corning, New-York, NY, USA), supplemented with 1% serum-free N-2 supplement (Gibco, Waltham, MA, USA), 2 mM glutamine, 15 mM HEPES (Gibco, Waltham, MA, USA), as well as 1 μg/mL tetracycline (Sigma-Aldrich, St. Louis, MO, USA), 1 mM dibutyryl cyclic-AMP (Sigma-Aldrich, St. Louis, MO, USA) and 10 ng/mL growth factor GDNF (Gibco, Waltham, MA, USA) at 37 °C in an atmosphere with 5% CO_2_. The medium was changed every two days. On the 7th day of cultivation, the differentiated LUHMES cells were used for research.

### 4.6. Staining of Vibratome Sections of the Mouse Substantia Nigra with GBR-BP 

Vibratome sections of the substantia nigra of adult mice were incubated for 1 h in KRB containing 50 nM GBR-BP at 37 °C. In the control, sections were preincubated for 10 min in KRB with 10 μM GBR-12909, and then 1 h in KRB with 10 μM GBR-12909 and 50 nM GBR-BP.

### 4.7. Staining of Living Cells of Primary Cultures of Mouse Mesencephalon and Metencephalon with GBR-BP and GBR-ATTO, as Well as Staining of LUHMES Cells with GBR-BP 

On the 7th day of cultivating the mouse mesencephalon and metencephalon, the medium was replaced with artificial cerebrospinal fluid (aCSF), containing 126 mM NaCl, 2.5 mM KCl, 1.2 mM NaH_2_PO_4_, 2.4 mM CaCl_2_, 1.2 mM MgCl_2_, 25 mM NaHCO_3_, 11 mM glucose, 0.15 mM ascorbic acid, and 20 mM HEPES (all reagents from Sigma-Aldrich, St. Louis, MO, USA) (pH 7.4) and incubation continued for 15 min to stabilize the cells. aCSF was used for all further incubations and for washing the cells. Four experiments were performed with mesencephalic cells (Figure 7B), in which the cells were incubated in an atmosphere of 5% CO_2_: (a) with 5 or 50 nM GBR-BP for 60 min, and in the control, first for 15 min with 10 μM GBR-12909, and then for 60 min with 10 μM GBR-12909 and 5 or 50 nM GBR-BP at 37 °C; (b) with 50 nM GBR-BP for 60 min at 4 °C or for the first 10 min with 450 mM sucrose, and for the next 60 min with 450 mM sucrose and 50 nm GBR-BP at 37 °C; (c) with 50 nM GBR-ATTO for 60 min, and in the control, first for 10 min with 10 μM GBR-12909, and then for 60 min with 10 μM GBR-12909 and 50 nM GBR-ATTO at 37 °C; (d) with 50 nM GBR-BP and 5 μM GBR-BP to assess the toxic effect 12 and 24 h later.

Metencephalic cells were incubated with only 50 nM GBR-BP for 60 min, and in the control, first for 10 min with 10 μM GBR-12909, and then for 60 min with 10 μM GBR-12909 and 50 nM GBR-BP at 37 °C (Figure 11C).

After differentiation, LUHMES cells were incubated at 37 °C in aCSF. The cells were stabilized for the first 15 min, and then two experiments were carried out (Figure 11D). In the first experiment, the cells were incubated for 1 h in aCSF containing 50 nM or 100 nM GBR-BP. As a control, in half of the wells (three wells with 1 × 105 cells per well), the cells were first incubated for 15 min in aCSF containing 10 μM GBR-12909, and in the control for 60 min in aCSF containing 10 μM GBR-12909 and 50 or 100 nM GBR-BP. In the second experiment, to assess the toxicity of GBR-BP, the cells were incubated for 12 h in a differentiation medium containing 50 nM or 5 μM GBR-BP with 0.05% DMSO. In the control, the cells were incubated in a differentiation medium containing 0.05% DMSO. After both experiments, the cells were washed three times for 5 min with aCSF and used for living cell microscopy.

To assess the duration of fluorescence of GBR-BP captured in monoaminergic neurons, we first incubated LUHMES cells with 50 nM GBR-BP for 1 h, washed them in aCSF, and incubated them in the differentiation medium. Cells were examined under a microscope 2, 24, 48, and 72 h after the end of incubation with GBR-BP.

### 4.8. Fixation and Immunostaining of Mouse Embryonic Mesencephalic and Metencephalic Cells, as Well as LUHMES Cells

In one case, immediately after the end of cultivation, and in the other case, after preliminary staining of living cells with GBR-BP, as well as in the control, after pre- and co-incubation with GBR-12909, mesencephalic and metencephalic cells were washed from aCSF with 0.02 M phosphate buffer with 0.9% NaCl (phosphate buffered saline, PBS, Gibco, Waltham, MA, USA) for 5 min at 20 °C. The cells were then fixed in 4% paraformaldehyde in 0.2 M phosphate buffer (pH 7.2–7.4) for 1 h at 4 °C. After being washed with PBS, the cells were incubated for 30 min at 20 °C in PBS with 3% bovine serum albumin (Sigma-Aldrich, St. Louis, MO, USA) and 0.3% Triton-X100 (Sigma-Aldrich, St. Louis, MO, USA). The cells were then incubated for 20 h at 20 °C in PBS containing the first antibodies (Table 1), 1% bovine serum albumin, and 0.1% Triton X-100. Mesencephalic and metencephalic cells were incubated in separate wells with one of the first antibodies: to TH (1:500), to DBH (1:500), and to 5-HT (1:750). The cells were then washed in PBS and incubated in PBS with the second fluorescent antibodies (Table 1). Finally, the cells were washed with PBS and embedded into the mounting medium with DAPI (Abcam, Cambridge, UK) for subsequent microscopy.

In another experiment, to detect antibodies to TH, biotinylated antibodies to sheep immunoglobulins (Vector Lab, Cambridge, UK) were also used. The cells were first incubated with these antibodies for 2 h at 20 °C, and then with horseradish peroxidase-containing avidin-biotin complex (1:1:100, Vector Lab, Cambridge, UK) for 1 h at 20 °C. Thereafter, the cells were washed in PBS, and peroxidase was revealed by incubation in a solution of 0.05% 3.3′-diaminobenzidine tetrahydrochloride and 0.03% H_2_O_2_. After the visualization of peroxidase, the cells were washed three times with PBS and embedded into the mounting medium with DAPI (Abcam, Cambridge, UK) for subsequent microscopy.

After differentiation, living LUHMES cells were washed in isotonic PBS solution for 5 min at 20 °C. The cells were then fixed in 4% paraformaldehyde in 0.2 M phosphate buffer (pH 7.2–7.4) for 1 h at 4 °C. After fixation, the cells were washed in PBS and incubated for 30 min at 20 °C in PBS containing 3% bovine serum albumin (Sigma-Aldrich, St. Louis, MO, USA) and 0.3% Triton-X100 (Sigma-Aldrich, St. Louis, MO, USA). After that, the cells were incubated for 20 h at 20 °C in PBS containing 1% bovine serum albumin, 0.1% Triton X-100, antibodies to TH (1:250) or antibodies to DAT (1:100), or antibodies to TH and DAT together. After incubation with primary antibodies, the cells were washed in PBS and incubated in PBS containing the secondary fluorescent antibodies for 2 h at 20 °C (Table 1). Finally, the cells were embedded into the mounting medium with DAPI (Abcam, Cambridge, UK) for subsequent microscopy.

To control the specificity of any immunolabeling used, primary antibodies were omitted.

### 4.9. Microscopy of Living Cells Stained with GBR-BP and GBR-ATTO, as Well as Microscopy of Fixed Immunostained Cells 

Microscopy of living cells, stained with GBR-BP and then fixed and immunostained, was performed using a Zeiss Observer Z1 microscope equipped with a Colibris 2.0 diode system (Zeiss, Oberkochen, Germany) and an AxioCam camera (Zeiss, Oberkochen, Germany) with Zeiss AxioVision software. The cells were examined using a 10× objective (EC PlnN 10×/0.3 DICI, Zeiss) and a 20× objective (Plan-Neufluar ×20/0.4, Zeiss).

**Figure 13:**
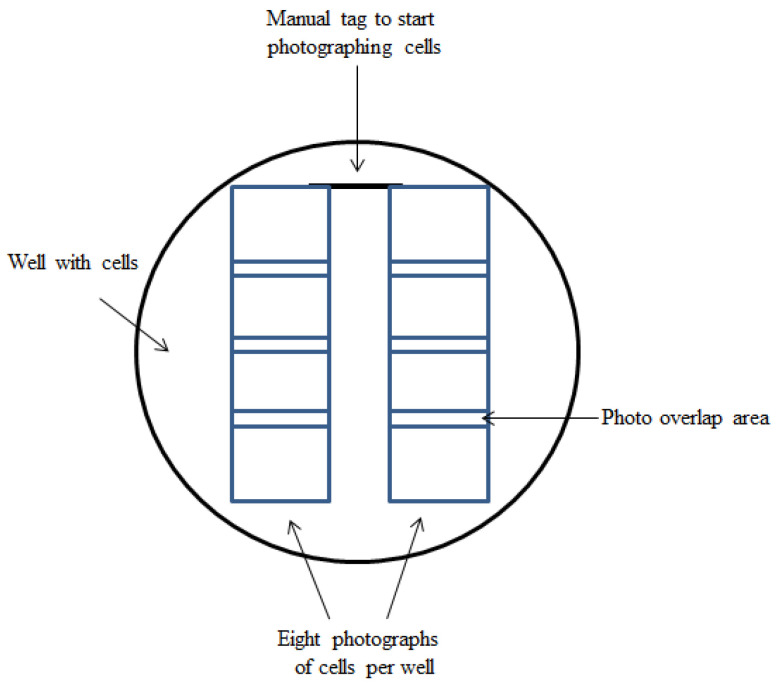
Scheme for obtaining eight photographs at 20× magnification in each well with a culture of mouse embryonic mesencephalic cells and LUHMES cells, first after staining living cells with GBR-BP, and then after fixing and immunostaining the same cells for the subsequent superposition of the respective images.

Fluorescence of GBR-BP was detected in the FITC channel (excitation filter 470/40, emission filter 530/50), while for GBR-ATTO the following parameters were used: excitation filter 550/25, emission filter 605/70. The scan start point was marked with a thin strip applied to the outside of the bottom of the well using a permanent marker (Edding 780, Tokyo, Japan). Scanning under a microscope was performed vertically relative to the applied mark (Figure 13). Cell images were also obtained in phase contrast.

For a more detailed assessment of the intracellular localization of GBR-BP, living cells were studied using a Leica SP5 laser confocal microscope (Leica, Wetzlar, Germany) with the LAS AF software (Leica, Wetzlar, Germany) and an immersion objective with a 63× magnification (HCX PL APO CS 63.0 × 1.40 OIL UV). Fluorescence of GBR-BP was excited by a laser with a wavelength of 488 nm and detected in the range from 502 to 536 nm. In some cases, living mesencephalic cells were photographed once per hour after incubation with 50 nM GBR-BP, or in the control after preincubation with 10 μM GBR-12909 and subsequent incubation with 10 μM GBR-12909 and 50 nM GBR-BP. In other cases, in the same experiment and in the control, time-lapse photography was carried out with an interval of 5 s for 17 min.

In each plate, LUHMES cells, mouse embryonic mesencephalic cells, and mouse embryonic metencephalic cells were photographed in five wells: in eight randomly selected fields of 0.14 mm² in each well at a 20× magnification (Plan-Neufluar ×20/0.4, Zeiss). In experiments with the mesencephalic cells, images obtained by photographing living cells stained with GBR-BP were superposed with images of the same cells, but after fixation and immunostaining. For this purpose, photography of living cells, first stained with GBR-BP, and then fixed and immunostained, was carried out in the same sectors of the wells, focusing on a previously made mark on the bottom of the well (Figure 12). After that, cells immunostained for one of the markers of monoaminergic neurons and cells stained with GBR-BP were counted.

In addition, we estimated the total number of cells in each primary culture of mouse mesencephalon and metencephalon by counting the number of DAPI-stained cell nuclei. Then, the number of TH-immunopositive and 5-HT-immunopositive cells in the mesencephalon culture and the number of DBH-immunopositive and 5-HT-immunopositive cells in the metencephalon culture were counted.

### 4.10. Long-Term Incubation of Mouse and Human Embryonic Mesencephalon Cells with GBR-BP

One week after the cultivation of the mouse embryonic mesencephalon and differentiation of LUHMES cells, GBR-BP was added to the incubation medium at a final concentration of 50 nM or 5 μM with 0.05% DMSO. In the control, only 0.05% DMSO was added to the incubation medium. The cells were incubated for 12 h at 37 °C and then washed with PBS. In each experiment and in the control, the cells were cultured in three wells (n = 3).

The subsequent processing of mouse embryonic mesencephalon cells and LUHMES cells was performed in significantly different ways. LUHMES cells were incubated for 30 min at 37 °C in a growth medium in the presence of 2 μM Calcein-AM and 0.5 μM Hoechst 33342 (Sigma-Aldrich, St. Louis, MO, USA) and then washed with PBS at the same temperature. Then, 500 μL of DMEM Fluorobright medium (Gibco, Waltham, MA, USA) was poured into each well, and photographs were taken of cells in 20 randomly selected areas 1.41375 × 10^−3^ cm^²^ in size (n = 20) in each of the wells.

Imaging was carried out in two channels at the same exposure and intensity in each well using a Zeiss Observer Z1 microscope equipped with a Colibris 2.0 diode system (Zeiss) and an AxioCam camera (Zeiss) with Zeiss AxioVision software using a 20× objective (Plan-Neufluar ×20/0.4). When shooting, the following filters were used: 365/525 nm for Hoechst-33342 and 475/525 nm for Calcein-AM.

After 12 h of incubation with 50 nM or 5 μM GBR-BP, the primary culture of the mouse embryonic mesencephalon was fixed with 4% paraformaldehyde in 0.2 M phosphate buffer for 60 min at 4 °C. Then we immunocytochemically identified TH-containing neurons using the primary and secondary antibodies linked to peroxidase (see Section 4.8).

In addition to the morphological quantitative analysis of changes in dopaminergic neurons in the mouse mesencephalon and differentiated LUHMES cells, we performed MTT assay (Sigma-Aldrich, St. Louis, MO, USA) of differentiated LUHMES cells in the same experiment, but with a longer incubation with GBR-BP (24 h). The differentiating medium with GBR-BP was replaced by the differentiating medium with MTT at a final concentration of 0.5 mg/mL, followed by incubation for 3.5 h at 37 °C. The incubation medium was then carefully removed and 200 μL DMSO was added to the LUHMES cells in each well for 10 min incubation at 37 °C. The purple extract was transferred to a 96-well plate. Optical absorption was measured on a plate reader at 540 nm (reference wavelength 630 nm).

### 4.11. Image Analysis

FiJi software (Fiji software. Available online: https://imagej.net/Fiji accessed on 11 February 2022) was used to assess neuronal loss (death) and neurite degradation. Images of cells stained with Calcein-AM were converted into the 8-bit format. After that, the grey threshold was set so that the stained neurites were displayed in grayscale, and the rest of the space remained black. After that, the “Area Fraction” function was used to calculate the area of the well surface covered by neurites. The number of cells was counted by converting images from Hoechst 33,342 into the 8-bit format. Next, we set the gray threshold in such a way that only the nuclei were colored, and the background remained black. The “Watershed” function was then used to separate the nuclei, which were “stuck together.” After that, the function “Analyze Particles” was used to count the cells.

To count the number of TH-immunopositive neurons, photographs were opened in the FiJi program. Immunopositive neurons were tagged in the photograph using the “Multi-Point” software function. After the cells were marked, the “Measure” software function was used to count them. In turn, neurites were manually drawn in its full length. Then, the length of the neurites of individual TH-immunopositive neurons stained with peroxidase was measured and their total length was estimated using the AxioVision software and the “Curve Spline” function.

The FiJi program was used to assess the fluorescence intensity of cells stained with GBR-BP at a concentration of 5 nM or 50 nM. The images were converted into the 8-bit forma and “Area”, “Integrated Density”, and “Mean Gray Value” were measured. After the required data were acquired, the fluorescence intensity was calculated using the following formula:

Corrected Total Cell Fluorescence (CTCF) = Integrated Density (IntDen) − (“Area of selected cell” × “Mean fluorescence of background values”).

### 4.12. Statistical Analysis

Data are presented as group mean ± standard error of mean. The correspondence of the data to the normal distribution was checked using the Shapiro–Wilk test. At a normal distribution (incubation with GBR-BP and control), the obtained data were treated statistically with Student’s *t*-test using GraphPad Prism 6.0 software (GraphPad Software, San Diego, CA, USA). At a non-normal distribution (other cases), the obtained data were treated statistically using a nonparametric one-way ANOVA test with Kruskal–Wallis criteria in the statskingdom.com software.

## 5. Conclusions

In this study, the following fundamentally important results were obtained:

(1) The fluorescent GBR-BP we synthesized on the GBR-12909 (DAT ligand) template is specifically internalized into the dopaminergic neurons of: (i) the mouse embryonic mesencephalon, (ii) the substantia nigra of adult mice, and (iii) human LUHMES cells via a DAT-dependent mechanism;
(2) In addition to dopaminergic neurons, GBR-BP is specifically internalized into some noradrenergic and serotonergic neurons of the mouse embryonic metencephalon, the anlage of the locus coeruleus, and the raphe nucleus, via membrane transporters;
(3) GBR-BP is nontoxic to the dopaminergic neurons of the mouse mesencephalon and LUHMES cells;
(4) The results obtained (points 1–3) open up broad prospects, on the one hand, for the visualization of living dopaminergic neurons for studying their functional activity in a mixed cell population, and, on the other hand, for the development of targeted drug delivery to dopaminergic neurons.

## Figures and Tables

**Table 1:** Primary and secondary antibodies used in these immunocytochemical studies.

Primary Antibodies		
**Antibody Target, Host.**	**Supplier**	**Dilution**
Anti-tyrosine hydroxylase, sheep	Chemicon, ab1542	1:500
Anti-tyrosine hydroxylase, mouse	Sigma, T1299	1:500
Anti-dopamine-ß-hydroxylase, sheep	Abcam, ab19353	1:500
Anti-5-hydroxytryptamine (serotonin), rabbit	Sigma, S5575	1:750
anti-DAT, rabbit	Abcam, ab111468	1:100
**Secondary Antibodies**		
Anti-gamma globulin of sheep, donkey, AlexaFluor 488	Invitrogen, A11015	1:700
Anti-gamma globulin of rabbit, donkey, AlexaFluor 546	Invitrogen, A10040	1:700
Anti-gamma globulin of sheep, rabbit, biotinylated	Vector Labs, BA-6000	1:200
ABC complex	Vector Labs	1:1:100

## Data Availability

The data presented in this study are available on request from the corresponding author. The data are not publicly available due to legal issues.

## Supplementary Materials

The following supporting information can be downloaded at: https://www.mdpi.com/article/10.3390/ijms23073678/s1.

## Abbreviations

5-HT	5-hydroxytryptamine
aCSF	artificial cerebrospinal fluid
ASP+	4-(4-(dimethylamino) styryl)-*N*-methylpyridinium iodide
BODIPY	4,4-difluoro-4-bora-3a,4a-diaza-s-indacene
Calcein-AM	Calcein O,O′-diacetate tetrakis(acetoxymethyl) ester
DAPI	4′,6-diamidino-2-phenylindole
DAT	dopamine transporter
DBH	dopamine-β-hydroxylase
DMEM/F12	Dulbecco’s Modified Eagle Medium and Nutrient Mixture F-12
DMSO	dimethyl sulfoxide
GBR-12909	1-(2-(bis(4-fluorophenyl)methoxy)ethyl)-4-(3-phenylpropyl)piperazine
GBR-BP	GBR-BODIPY
HEPES	2-[4-(2-hydroxyethyl)piperazin-1-yl]ethanesulfonic acid
HPLC	high performance liquid chromatography
KRB	Krebs-Ringer buffer
LUHMES	Lund human embryonic mesencephalic
MPP+	1-methyl-4-phenylpyridinium iodide
MTT	3-(4,5-dimethyl-2-thiazolyl)2,5-diphenyl-2*H*-tetrazolium bromide
PBS	phosphate-buffered saline
PD	Parkinson’s disease
TFA	trifluoroacetic acid
TH	tyrosine hydroxylase
TLC	Thin-layer chromatography
UV	ultraviolet
