# Supplementary material for: A New Method for the Visualization of Living Dopaminergic Neurons and Prospects for Using It to Develop Targeted Drug Delivery to These Cells
Source: Int J Mol Sci. 2022 Mar 27;23(7):3678. doi: 10.3390/ijms23073678 (PMC8998426; doi:10.3390/ijms23073678)
Supplement: Supplementary file 1 [file ijms-23-03678-s001.zip › Supplements Blokhin Lavrova Ugrumov_v3.pptx]

## Slide 1
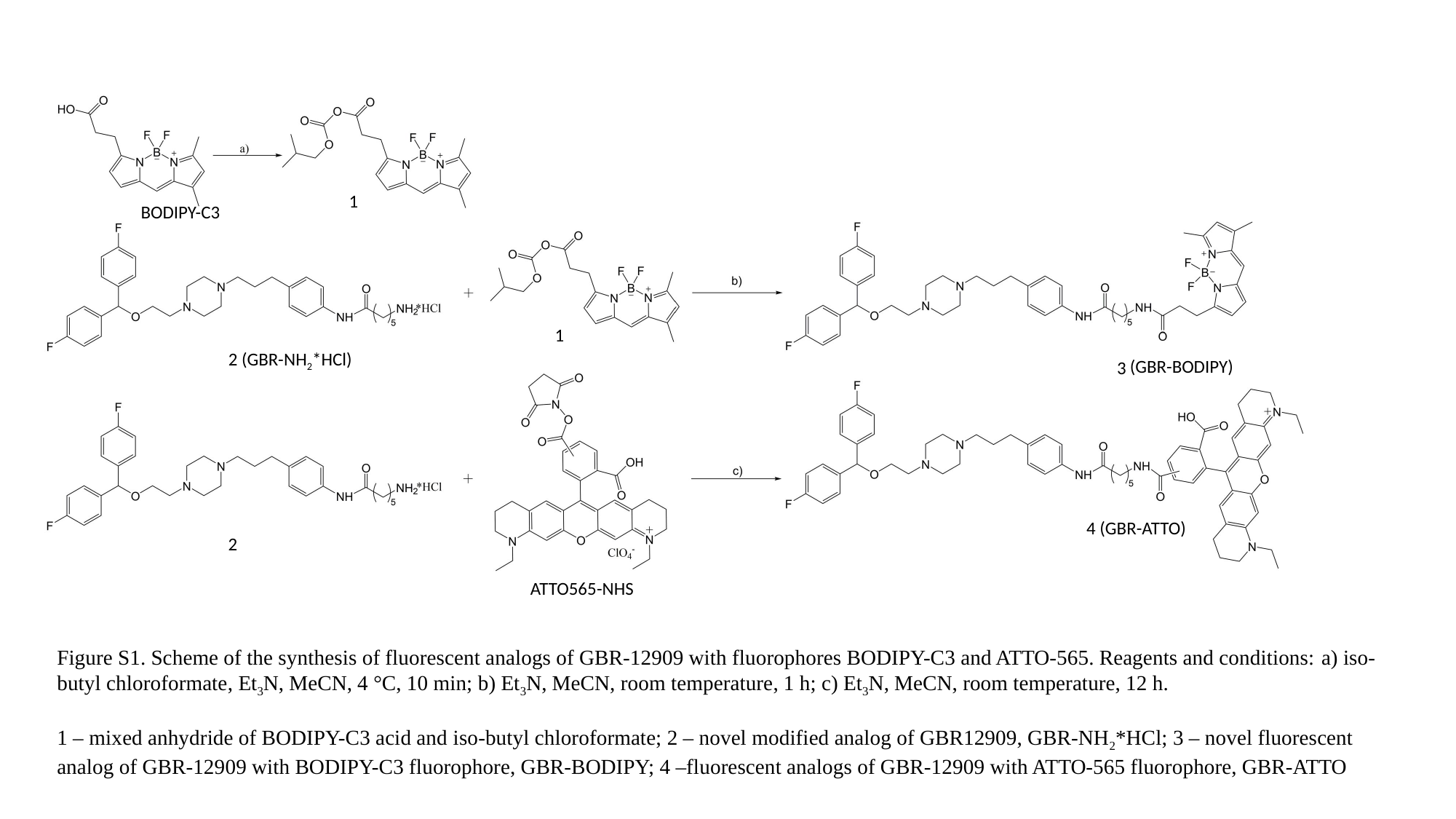

1
BODIPY-C3
1
2 (GBR-NH2*HCl)
(GBR-BODIPY)
3
4 (GBR-ATTO)
2
ATTO565-NHS
Figure S1. Scheme of the synthesis of fluorescent analogs of GBR-12909 with fluorophores BODIPY-C3 and ATTO-565. Reagents and conditions: a) iso-butyl chloroformate, Et3N, MeCN, 4 °C, 10 min; b) Et3N, MeCN, room temperature, 1 h; c) Et3N, MeCN, room temperature, 12 h.
1 – mixed anhydride of BODIPY-C3 acid and iso-butyl chloroformate; 2 – novel modified analog of GBR12909, GBR-NH2*HCl; 3 – novel fluorescent analog of GBR-12909 with BODIPY-C3 fluorophore, GBR-BODIPY; 4 –fluorescent analogs of GBR-12909 with ATTO-565 fluorophore, GBR-ATTO

## Slide 2
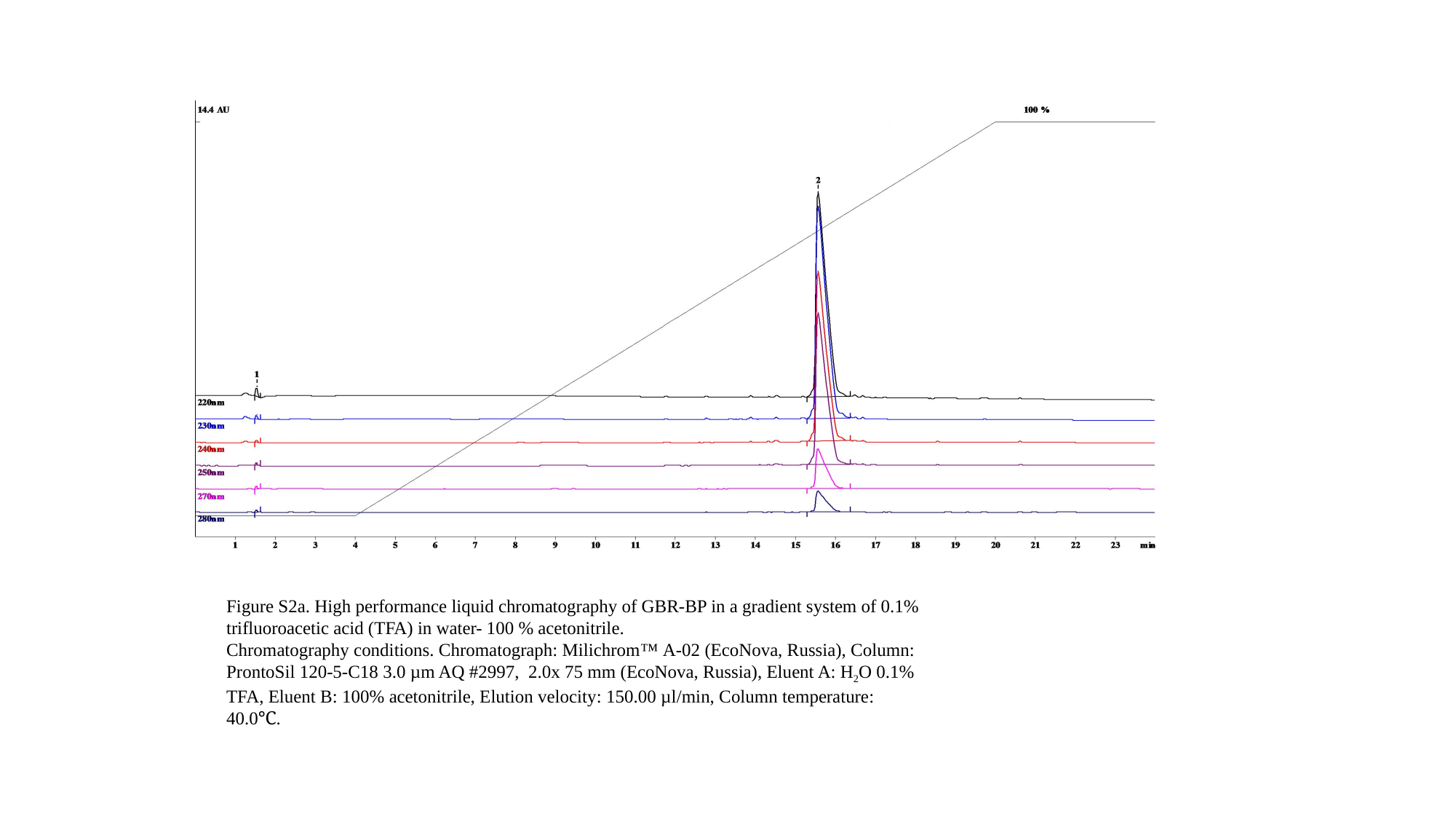

Figure S2a. High performance liquid chromatography of GBR-BP in a gradient system of 0.1% trifluoroacetic acid (TFA) in water- 100 % acetonitrile.
Chromatography conditions. Chromatograph: Milichrom™ А-02 (EcoNova, Russia), Column: ProntoSil 120-5-C18 3.0 µm AQ #2997, 2.0x 75 mm (EcoNova, Russia), Eluent A: H2O 0.1% TFA, Eluent B: 100% acetonitrile, Elution velocity: 150.00 µl/min, Column temperature: 40.0℃.

## Slide 3
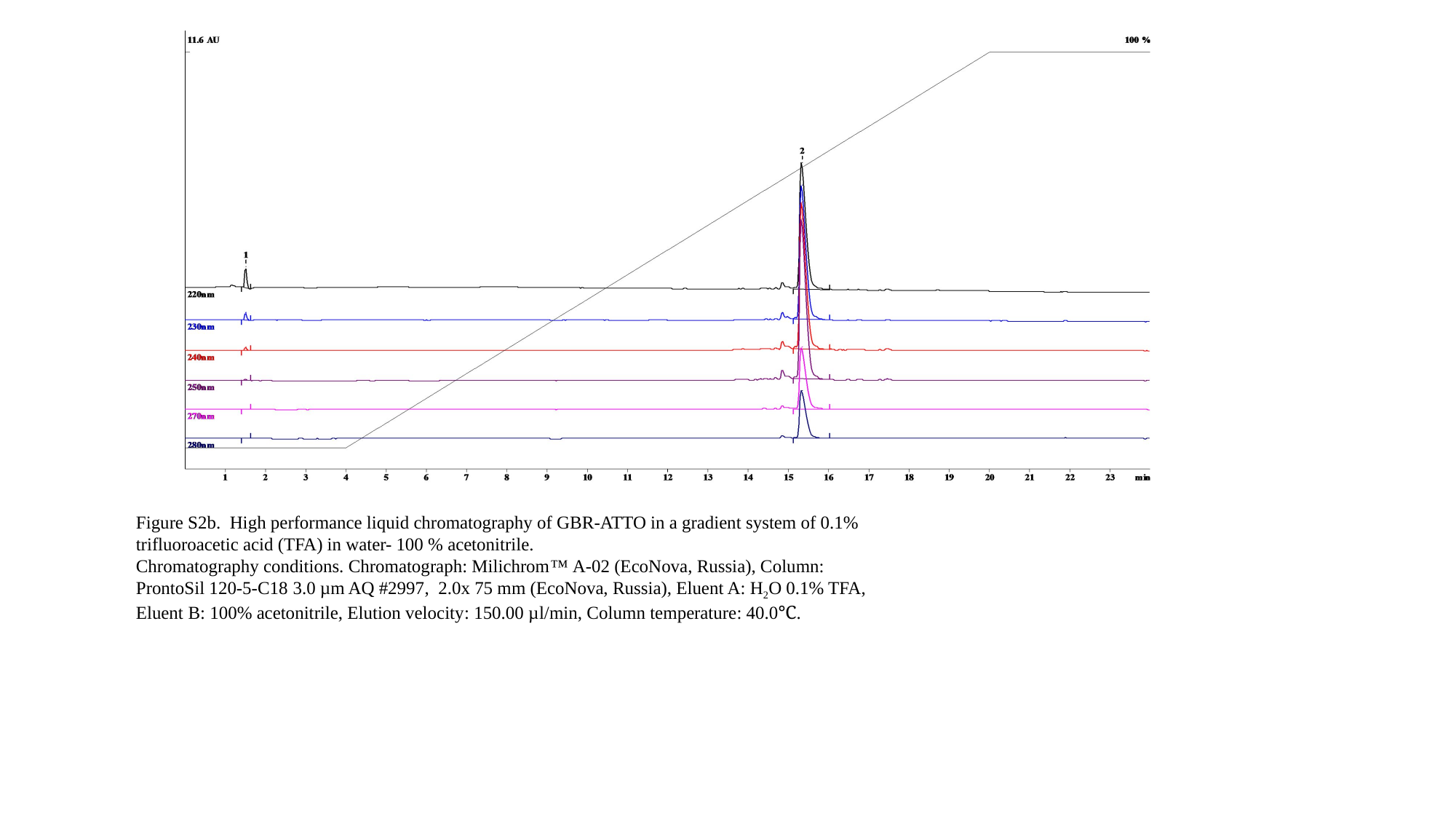

Figure S2b. High performance liquid chromatography of GBR-ATTO in a gradient system of 0.1% trifluoroacetic acid (TFA) in water- 100 % acetonitrile.
Chromatography conditions. Chromatograph: Milichrom™ А-02 (EcoNova, Russia), Column: ProntoSil 120-5-C18 3.0 µm AQ #2997, 2.0x 75 mm (EcoNova, Russia), Eluent A: H2O 0.1% TFA, Eluent B: 100% acetonitrile, Elution velocity: 150.00 µl/min, Column temperature: 40.0℃.

## Slide 4
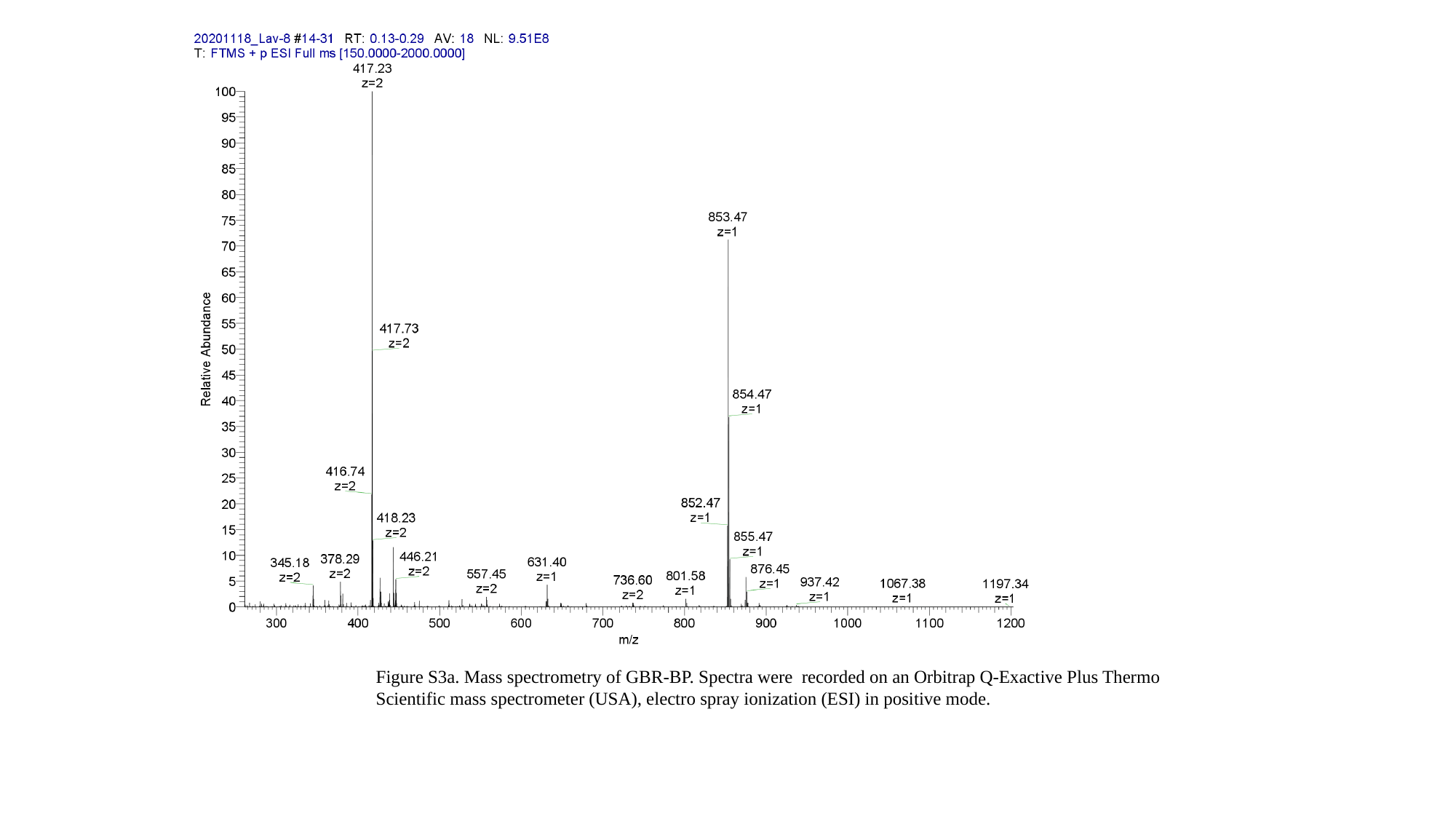

Figure S3a. Mass spectrometry of GBR-BP. Spectra were recorded on an Orbitrap Q-Exactive Plus Thermo Scientific mass spectrometer (USA), electro spray ionization (ESI) in positive mode.

## Slide 5
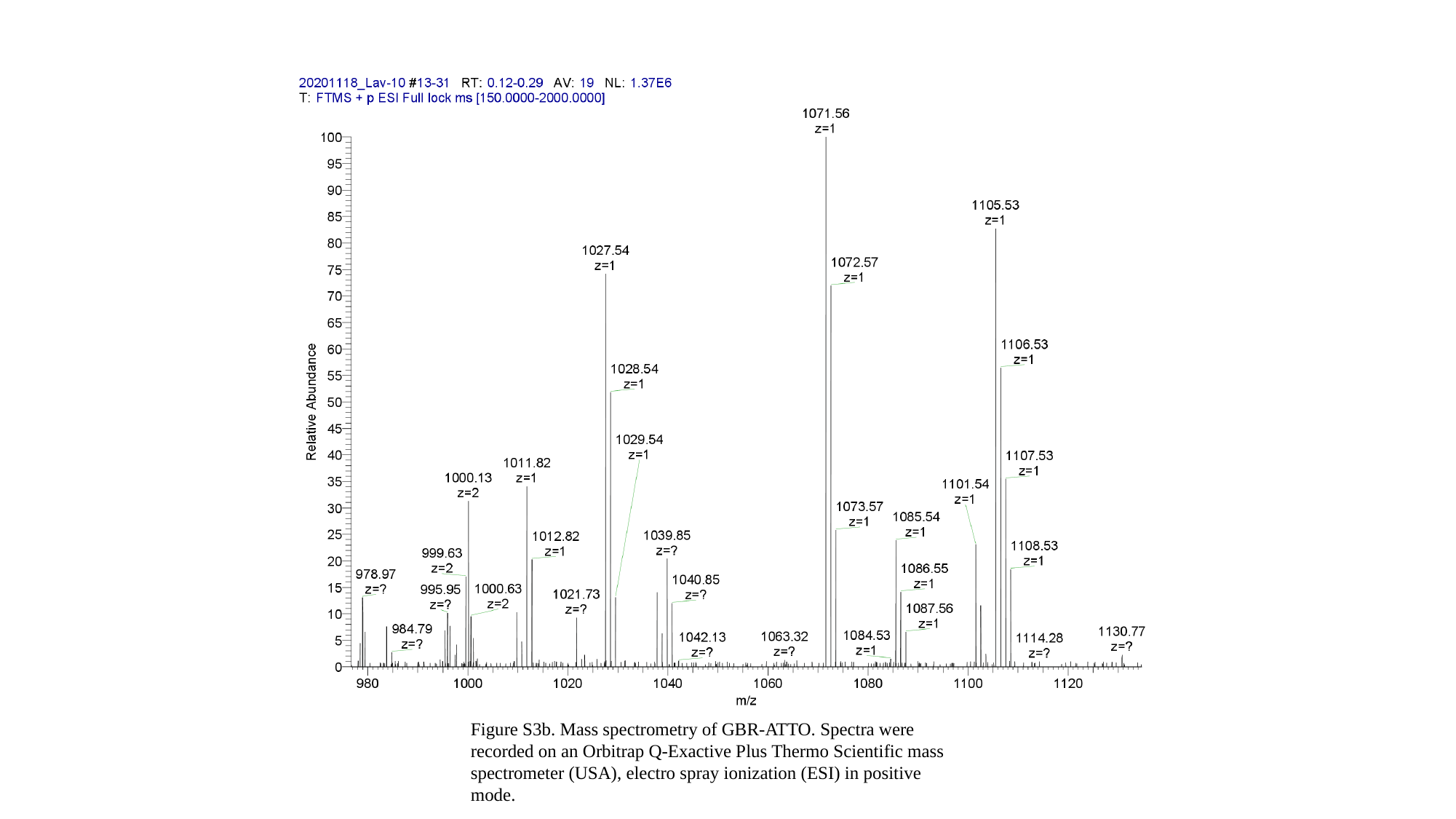

Figure S3b. Mass spectrometry of GBR-ATTO. Spectra were recorded on an Orbitrap Q-Exactive Plus Thermo Scientific mass spectrometer (USA), electro spray ionization (ESI) in positive mode.

## Slide 6
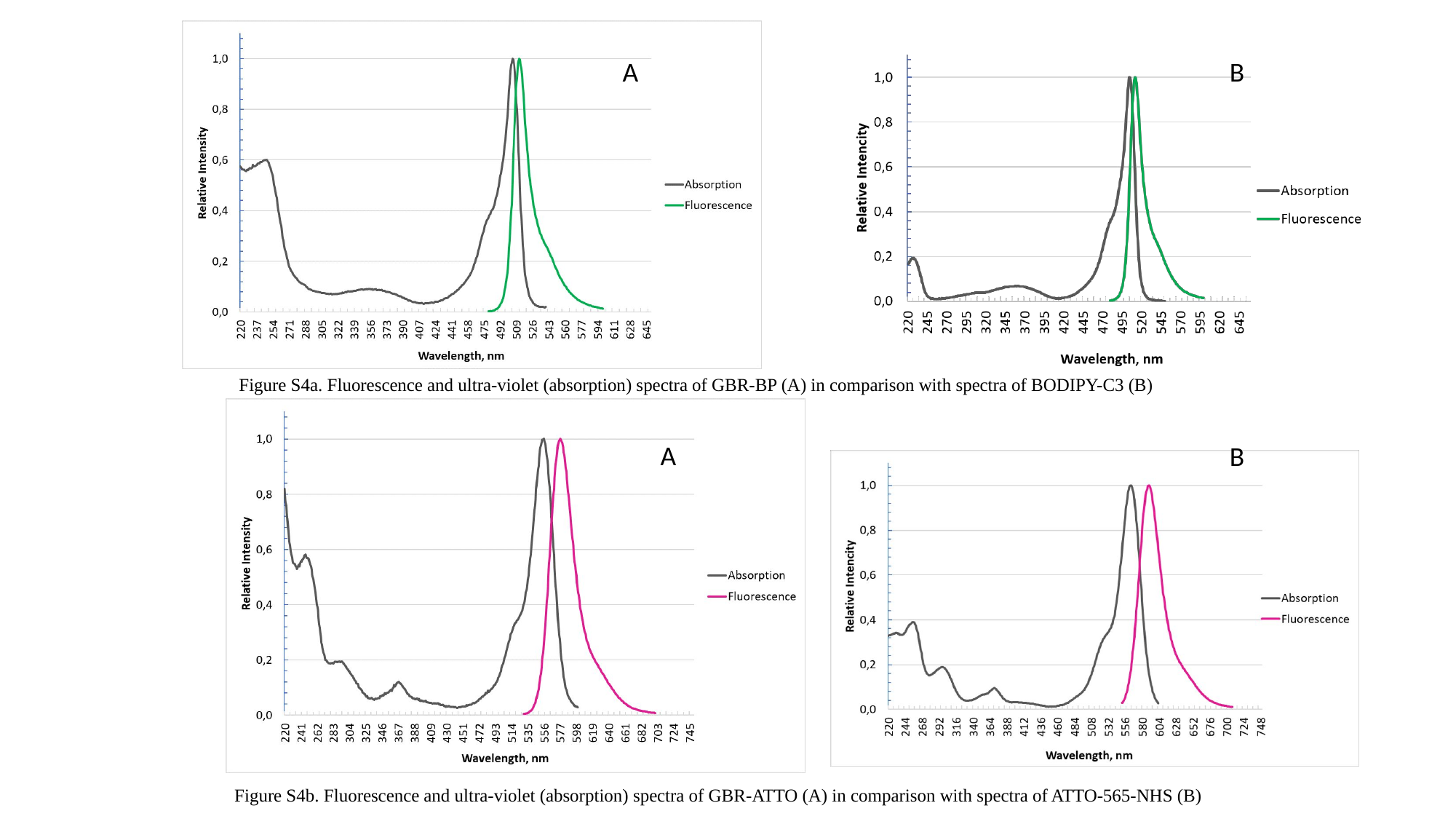

A
B
 Figure S4a. Fluorescence and ultra-violet (absorption) spectra of GBR-BP (A) in comparison with spectra of BODIPY-C3 (B)
A
B
Figure S4b. Fluorescence and ultra-violet (absorption) spectra of GBR-ATTO (A) in comparison with spectra of ATTO-565-NHS (B)

## Slide 7
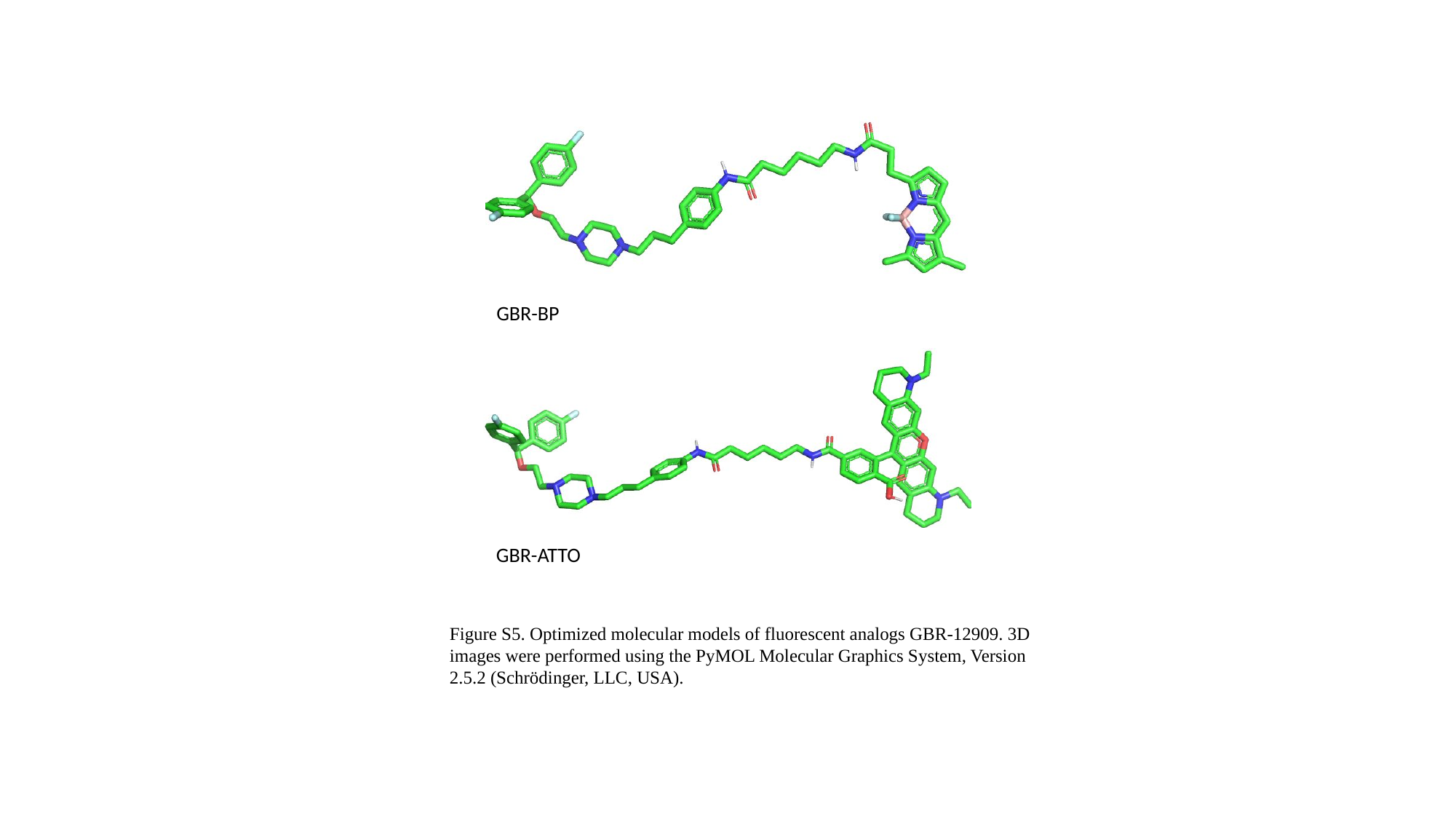

GBR-BP
GBR-ATTO
Figure S5. Optimized molecular models of fluorescent analogs GBR-12909. 3D images were performed using the PyMOL Molecular Graphics System, Version 2.5.2 (Schrödinger, LLC, USA).
